# Efficient CRISPR‐Cas9 delivery and transgene‐free multiplex genome editing in plants using cymbidium mosaic virus‐derived vectors

**DOI:** 10.1111/tpj.71031

**Published:** 2026-07-06

**Authors:** Ying‐Wen Huang, Chung‐Chi Hu, Yu‐Hsiu Cho, Ching‐Hsiu Tsai, Na‐Sheng Lin, Yau‐Heiu Hsu, Savithramma P. Dinesh‐Kumar

**Affiliations:** ^1^ Graduate Institute of Biotechnology, National Chung Hsing University Taichung 40227 Taiwan; ^2^ Advanced Plant and Food Crop Biotechnology Center National Chung Hsing University Taichung 40227 Taiwan; ^3^ Biology Department National Museum of Natural Science Taichung Taiwan; ^4^ Institute of Plant and Microbial Biology, Academia Sinica Taipei 11529 Taiwan; ^5^ Department of Plant Biology and The Genome Center The College of Biological Sciences, University of California Davis California 95616 USA; ^6^ Innovative Genomics Institute, University of California, Berkeley Berkeley California 94720 USA

**Keywords:** CRISPR‐Cas9, virus‐induced genome editing, cymbidium mosaic virus, *Nicotiana benthamiana*, *Phalaenopsis aphrodite*

## Abstract

Virus‐induced genome editing (VIGE) has become a useful method by enabling transient delivery of gene‐editing reagents; however, many viral systems face limitations in cargo size, host range, or reliance on transgenic Cas9‐expressing plants. In this study, we developed a cymbidium mosaic virus (CymMV)‐based VIGE platform that enables simultaneous expression of *Streptococcus pyogenes* Cas9 (SpCas9) and one or more guide RNAs (gRNAs) from a single viral RNA. In *Nicotiana benthamiana*, this system induced editing in the *Phytoene desaturase* (*PDS*) gene, with indel rates exceeding 50% within 6 days after inoculation, outperforming traditional delivery methods by about fivefold. Notably, over 80% of regenerated plants contained targeted mutations, and 82% of these were both transgene‐ and virus‐free, including tetra‐allelic knockouts directly in the M0 generation. Adding a Ruby‐based visual counterselection marker enabled rapid, reliable identification of transgene‐free, edited plants without antibiotic selection. When adapted to *Phalaenopsis aphrodite* orchids, the platform efficiently edited the *PaPDS* gene, achieving a 47% indel frequency at 20 days post‐inoculation, with visible bleaching in leaf tissue from inoculated protocorm‐like bodies. Additionally, expressing multiple gRNAs from a single CymMV replicon enabled multiplex editing in orchid tissues, demonstrating the system's versatility for complex, polyploid crops. Our findings broaden the use of VIGE in orchids and provide a reliable framework for precision plant breeding.

## Significance Statement

Efficient genome editing remains technically challenging in orchids, limiting both functional studies and precision breeding in this important horticultural group. Here, we establish a cymbidium mosaic virus‐based CRISPR‐Cas9 delivery platform that enables targeted mutagenesis in *Phalaenopsis aphrodite* and provides a practical framework for orchid genome engineering.

## INTRODUCTION

Precise and efficient genome editing is now essential for functional genomics and crop improvement in plants. Among the available technologies, CRISPR‐Cas9 has enabled targeted mutations in many plant species (Chen et al., 2019; Zhu et al., 2020). It works by guiding the *Streptococcus pyogenes* Cas9 nuclease to a specific genomic site using a guide RNA (gRNA) that matches a 20‐nucleotide sequence next to a protospacer adjacent motif (PAM, with the nucleotide sequence of 5′‐NGG‐3′). Cas9 then creates a double‐strand break (DSB) at this location. The cell repairs this break mainly through non‐homologous end joining (NHEJ), which can introduce insertions or deletions (Indels), potentially knocking out or modifying gene functions (Doudna & Charpentier, 2014; Jinek et al., 2012). Compared to traditional breeding, CRISPR‐Cas9 allows faster and more precise gene editing with fewer unintended mutations (Mao et al., 2019; Wang et al., 2022). Despite its advancements, genome editing in most plants still relies on methods such as *Agrobacterium*‐mediated transformation or particle bombardment to deliver Cas9 and gRNA into the genome, thereby producing stable transgenic plants (Altpeter et al., 2016; Han et al., 2025). Residual transgenes can pose concerns regarding genome stability, off‐target effects, and regulatory issues for genetically modified organisms (Voytas & Gao, 2014). To produce transgene‐free edited plants, the typical approach involves breeding to segregate out the transgenes through selfing or backcrossing. This process is labor‐intensive and slow, especially in vegetatively propagated species or highly heterozygous hybrids where segregation of foreign DNA can be difficult (He & Zhao, 2020).

Virus‐induced genome editing (VIGE) presents a promising alternative to traditional transgene‐based methods for creating transgene‐free plant genomes. By utilizing engineered plant viral genomes as delivery carriers for genome editing molecules, VIGE allows for the temporary introduction of Cas9 and gRNAs without permanently integrating them into the plant's DNA (Oh et al., 2021; Shen et al., 2024; Zhang et al., 2022). Since plant viruses replicate episomally and can spread throughout the plant systemically, VIGE provides an efficient means of genome editing, especially in species that are challenging to modify with standard transformation techniques (Chang & Ku, 2024; Ma et al., 2020; Mikhaylova, 2025; Uranga & Daròs, 2023).

Although promising, the widespread use of VIGE is limited by the small cargo capacity of most plant viruses (Steinberger & Voytas, 2025). The sizable Cas9 gene (~4.1 kb) often surpasses what viral genomes can carry, so many VIGE systems only deliver gRNAs into existing Cas9 transgenic plants instead of editing directly in wild‐type plants (Ali et al., 2015; Baysal et al., 2025; Ellison et al., 2020; Kang et al., 2025; Nagalakshmi et al., 2022; Oh et al., 2025; Yin et al., 2015). Additionally, plant viruses tend to have restricted host ranges, and their ability to infect and spread varies widely across plant species, which limits the transferability of VIGE systems across crops (Varanda et al., 2021). Consequently, creating host‐specific viral vectors is becoming a vital strategy to overcome these challenges and expand the use of VIGE technology across various plants (Wu et al., 2024).

Cymbidium mosaic virus (CymMV) is a positive‐sense single‐stranded RNA (+ssRNA) virus belonging to the *Potexvirus* genus. It is one of the most common viruses infecting orchids and has been studied extensively at the molecular level. CymMV‐based vectors have been created for virus‐induced gene silencing (VIGS) and transient gene expression in orchids (Hsieh et al., 2013; Lee et al., 2021), highlighting their potential as tools for functional genomics in ornamental plants. However, it remains unclear whether CymMV can be reliably adapted as a VIGE platform to enable efficient CRISPR/Cas9 genome editing, as this has not been systematically investigated.

In this study, we developed a CymMV‐based CRISPR‐Cas9 genome editing system in which Cas9 and gRNA are concurrently delivered and co‐expressed from a single viral replicon. We tested its effectiveness in a model plant and an orchid species. Through optimizing the vector design and expression methods, we show that CymMV enables efficient genome editing and offers a flexible platform for transgene‐free plant genome modification.

## RESULTS

### Development and characterization of a CymMV‐based CRISPR‐Cas9 system for efficient gene editing in *Nicotiana benthamiana*

**Figure 1:**
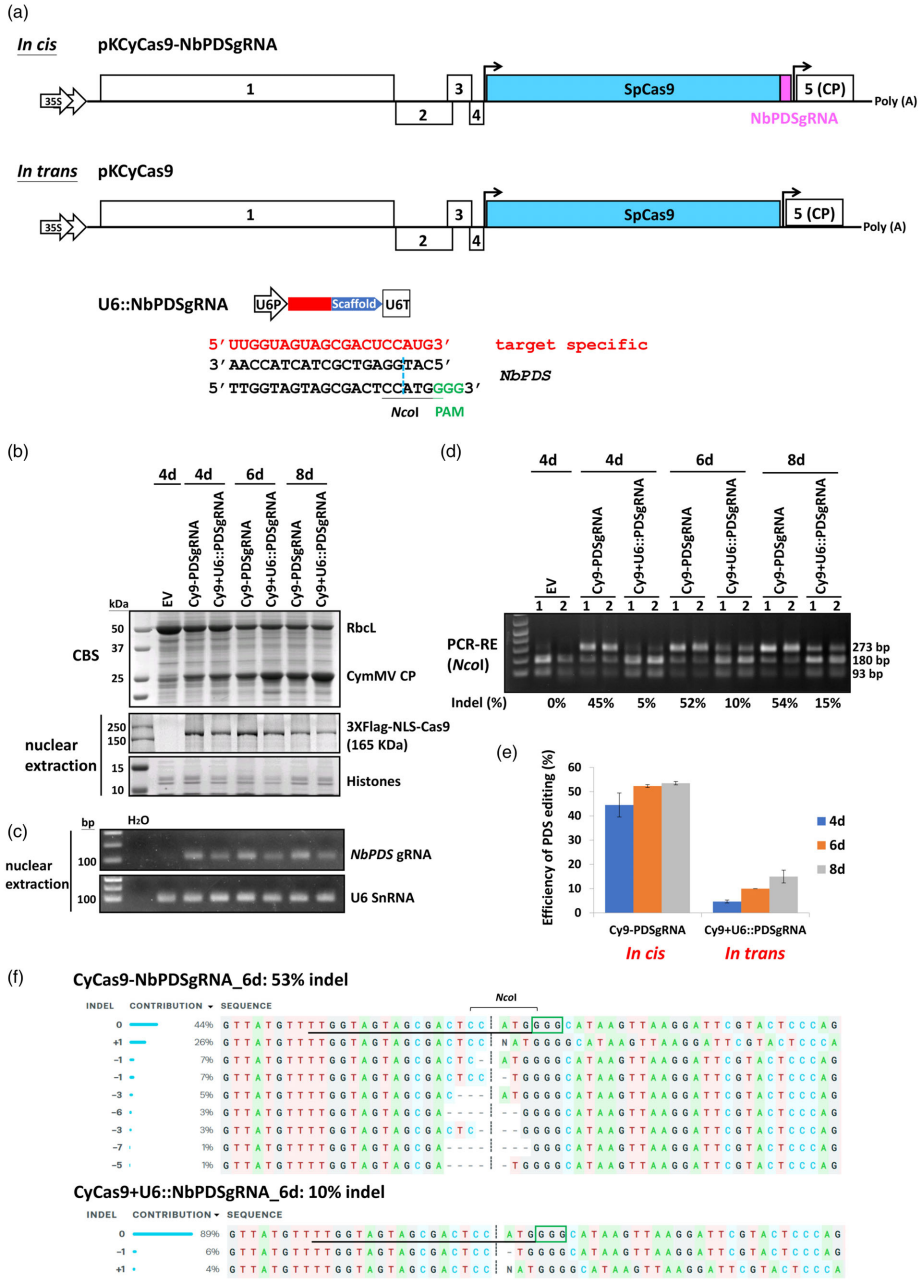
Development and optimization of cymbidium mosaic virus (CymMV)‐based CRISPR/Cas9 delivery systems in *Nicotiana benthamiana*. (a) Schematic diagrams of viral vectors used for *in cis* and *in trans* delivery strategies. In the *in cis* design (pKCyCas9‐NbPDSgRNA), the gRNA is driven by a duplicated coat protein (CP) subgenomic promoter (indicated by arrows) and expressed from the same vector as Cas9. In the *in trans* design, Cas9 is expressed from the CymMV vector (pKCyCas9), while the gRNA is expressed separately from a U6 promoter (U6::NbPDSgRNA). (b) Coomassie Blue‐stained (CBS) SDS‐PAGE gel showing total protein profiles and nuclear extracts at 4, 6, and 8 days post‐infiltration (dpi). Bands corresponding to the Rubisco large subunit (RbcL), CymMV CP, and Cas9 (3XFlag‐NLS‐Cas9) are indicated. Histone proteins were used as markers of the nuclear fraction. EV, empty vector (pKn). (c) RT‐PCR detection of NbPDS gRNA accumulation in the nuclear fraction of infiltrated leaves. U6 snRNA served as an internal control. (d) Comparison of editing efficiencies between *in cis* and *in trans* strategies using PCR‐RE (*Nco*I digestion) assays. The presence of undigested bands (273 bp) indicates indels. Indel percentages, calculated by Inference of CRISPR Edits (ICE) analysis, are shown below the gels. (e) Quantification of *NbPDS* editing efficiency in *in cis* and *in trans* designs at different time points. Data represent mean ± SD from three independent biological replicates. (f) Representative Sanger sequencing and ICE analysis results of the *NbPDS* target region from leaves infected with the *in cis* and *in trans* vectors at 6 dpi, showing diverse indel patterns.

CymMV has a +ssRNA genome with five major open reading frames encoding the viral RNA‐dependent RNA polymerase (RdRp), three movement‐associated proteins known as the triple gene block proteins (TGBp1, 2, and 3), and the coat protein (CP) (Figure 1a, top) (Wong et al., 1997). In addition to replicating the full‐length genomic RNA, CymMV produces a set of subgenomic RNAs that enable expression of downstream viral proteins, among which the CP subgenomic promoter (SGP) is the strongest. In this study, we engineered a CymMV‐based vector by duplicating the CP SGP to drive SpCas9 expression (Figure 1a).

To determine the optimal expression strategy for Cas9 delivery using CymMV, we compared genome‐editing efficiencies between *cis* and *trans* configurations in *N. benthamiana* by targeting the *Phytoene desaturase* (*NbPDS*) gene (Figure 1a). *Nicotiana benthamiana* is an allotetraploid species with two conserved *NbPDS* homoeologs (*NbPDS‐A* and *NbPDS‐B*); therefore, a gRNA was designed to target both loci simultaneously using an identical protospacer and PAM sequence (Figure S1). Two viral vector strategies were employed for comparison: (i) an *in cis* configuration, in which the gRNA sequence was inserted immediately downstream of the Cas9 stop codon (pKCyCas9‐NbPDSgRNA) (Figure 1a, top); and (ii) an *in trans* configuration, in which Cas9 is expressed from the CymMV vector (pKCyCas9) while the gRNA is expressed separately under a plant U6 promoter (U6::NbPDSgRNA) (Figure 1a, bottom).

To determine whether Cas9 and gRNA are expressed, *Agrobacterium* harboring pKCyCas9‐NbPDSgRNA or a mixture of *Agrobacterium* harboring pKCyCas9 and U6:NbPDSgRNA was infiltrated into 4‐week‐old *N. benthamiana* leaves. Nuclear extracts were prepared from infiltrated leaves at 4, 6, and 8 days post‐infiltration (dpi). Immunoblot analysis with anti‐FLAG antibodies detected Cas9 protein in both CyCas9‐NbPDSgRNA‐infiltrated and CyCas9/U6:NbPDSgRNA co‐infiltrated samples at 4, 6, and 8 dpi (Figure 1b). Furthermore, RT‐PCR detected NbPDS gRNA (Figure 1c). To determine whether this nuclear gRNA‐associated signal could be attributed to longer viral genomic or subgenomic RNA transcripts, we performed additional RT‐PCR analysis targeting upstream Cas9‐linked viral RNA regions using total RNA and nuclear‐enriched RNA fractions (Figure S2). These upstream viral RNA regions were detected in the total RNA fraction but not in the nuclear‐enriched fraction (Figure S2b, third panel), suggesting that the gRNA‐associated RNA species detected in the nuclear‐enriched fraction were unlikely to originate from intact viral RNA transcripts. Together, these results indicate that the CymMV vector can be used to efficiently express Cas9 and gRNA in both *cis* and *trans* configurations.

We next assessed genome‐editing activity using PCR‐restriction digestion (PCR‐RE) and sequence analysis. The region flanking the *NbPDS* target, containing the *Nco*I restriction site, was amplified by PCR and digested with *Nco*I. Indel mutations that disrupted the *Nco*I site generated digestion‐resistant fragments at higher efficiency in CyCas9‐NbPDSgRNA‐infiltrated samples than in CyCas9/U6:NbPDSgRNA co‐infiltrated samples (Figure 1d). Sanger sequencing followed by Inference of CRISPR Edits (ICE) analysis (Conant et al., 2022) showed 45, 52, and 54% indels in CyCas9‐NbPDSgRNA‐infiltrated samples at 4, 6, and 8 dpi (Figure 1d–f). In CyCas9/U6:NbPDSgRNA co‐infiltrated samples, the indel efficiency was 5, 10, and 15% at 4, 6, and 8 dpi (Figure 1d–f). These results indicate that the *in cis* configuration induces higher editing efficiency than the *in trans* configuration.

To determine whether the *in cis* configuration is preferred even in a non‐viral transient expression system, Cas9 and the NbPDS gRNA were co‐expressed either from separate transcriptional units (*in trans*) or from a single unit (*in cis*) under the control of the 35S promoter (Figure S3a). At 4 dpi, the single *in cis* unit produced approximately threefold higher indel frequencies than the *in trans* configuration (Figure S3b–d). These results demonstrate that coordinated expression of Cas9 and gRNA from a single transcriptional unit enhances editing efficiency independently of viral replication, further supporting the intrinsic advantages of the *in cis* design.

Together, these results demonstrate that CymMV has sufficient cargo capacity to carry the Cas9 sequence and that co‐expression of Cas9 and gRNA in a *cis* configuration markedly enhances genome editing efficiency in infiltrated leaves, supporting its use as a core design principle for CymMV‐based VIGE applications.

### A CP‐deleted CymMV replicon supports efficient Cas9 expression and genome editing

**Figure 2:**
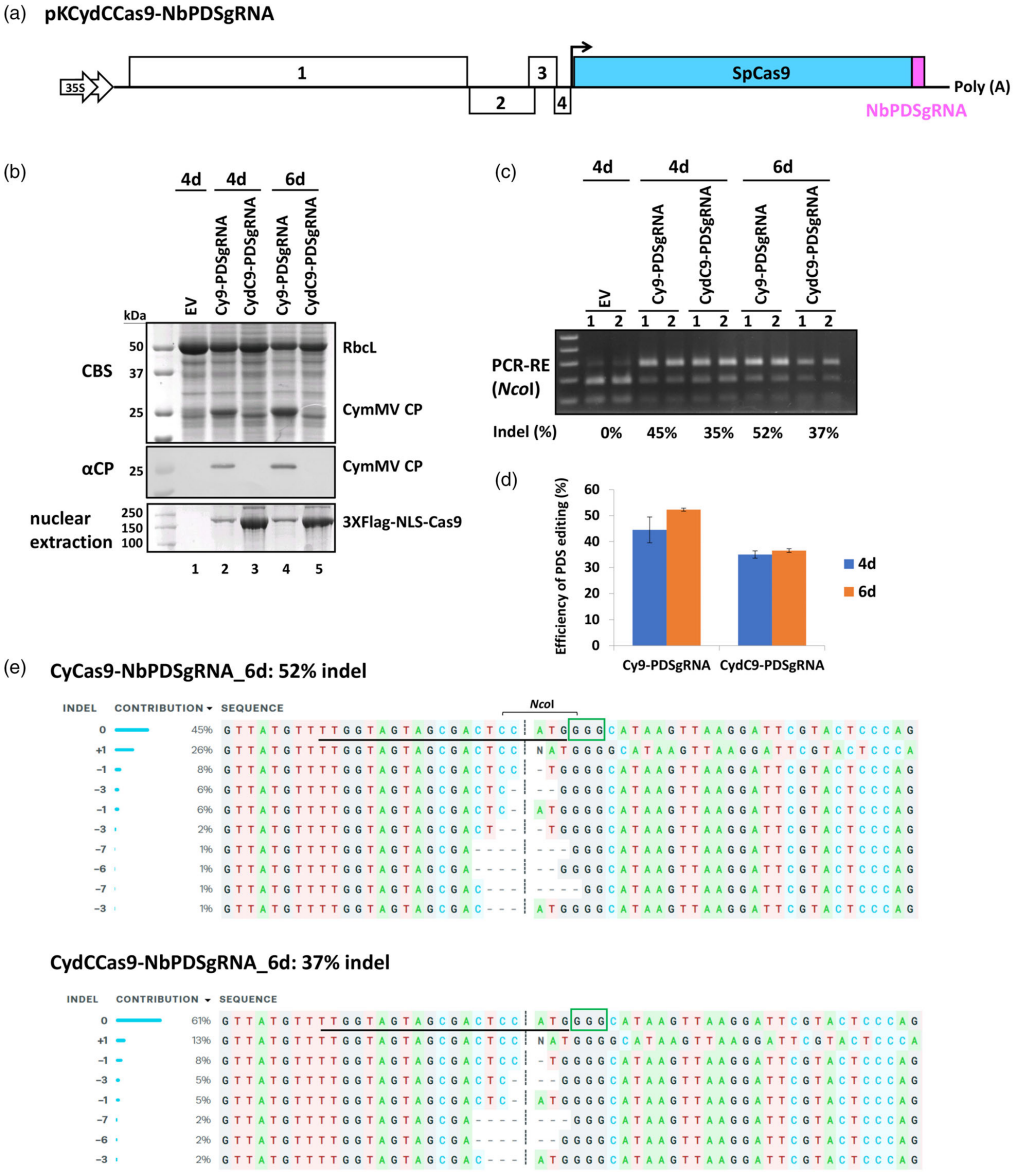
Assessment of a movement‐defective cymbidium mosaic virus (CymMV) vector for genome editing. (a) Schematic of the coat protein (CP)‐deficient viral vector (pKCydCCas9‐NbPDSgRNA). (b) Coomassie Blue‐stained (CBS) SDS‐PAGE and immunoblot analysis showing CymMV CP accumulation and Cas9 expression at 4 and 6 dpi. (c) PCR‐RE analysis of *NbPDS* editing efficiency mediated by full‐length CymMV and CP‐deleted CymMV replicon. (d) Quantification of indel frequencies comparing the full‐length and movement‐defective vectors at the indicated time points. (e) Sequence analysis of mutations induced by the CydC vector, confirming efficient somatic editing.

To improve biosafety and limit viral dissemination, we generated a CP‐deleted CymMV vector that functions as a movement‐defective viral replicon (Figure 2a). CymMV belongs to the genus *Potexvirus*, in which the CP, together with the TGBps, is required for efficient cell‐to‐cell movement and systemic infection (Lu et al., 2009; Verchot‐Lubicz, 2005). Thus, deletion of the CP coding region is expected to abolish virion formation and severely restrict viral spread while retaining viral replication competence and subgenomic RNA expression within initially infected or agroinfiltrated tissues. Immunoblot analyses of nuclear extracts from samples at 4 and 6 dpi revealed significantly increased Cas9 protein expression in CP‐deleted CymMV compared to full‐length CymMV (Figure 2b, lanes 3 versus 2 and lanes 5 versus 4). PCR‐RE and ICE analysis showed 35–37% editing efficiency at the *PDS* locus with CP‐deleted CymMV, compared with 45–52% with full‐length CymMV (Figure 2c–e). These results suggest that increased Cas9 expression using CP‐deleted CymMV does not translate into increased editing efficiency. Even so, the CP‐deleted CymMV replicon remained capable of mediating efficient indel formation while incorporating an intrinsic biocontainment feature through the loss of viral movement. This balance between editing efficiency and biosafety concerns may highlight the suitability of movement‐defective CymMV replicons as practical and controllable platforms for plant genome engineering.

### CymMV‐mediated VIGE enables efficient, heritable, transgene‐ and virus‐free editing in *N. benthamiana*

**Figure 3:**
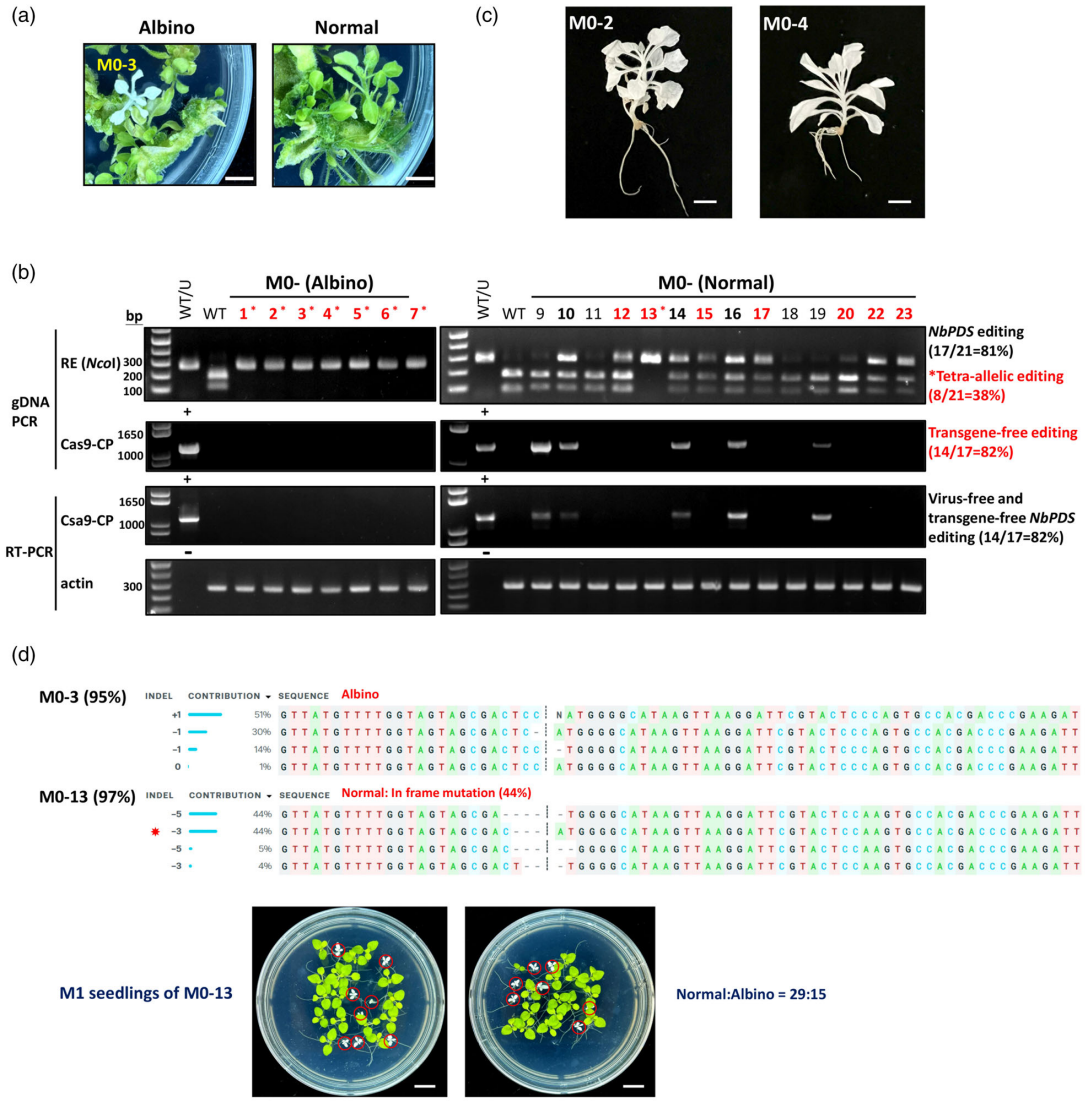
Regeneration and genotyping of transgene‐free and virus‐free edited plants. (a) Representative regenerated shoots from infected leaf explants, showing albino and normal phenotypes. (b) Molecular characterization of regenerated M0 lines from cymbidium mosaic virus (CymMV)‐infected leaf explants (6 dpi). PCR‐RE analysis was performed to detect mutations at the *NbPDS* locus. Sample numbers in bold indicate lines carrying detectable *NbPDS* editing. Red sample numbers indicate transgene‐free lines. Red asterisks (*) indicate lines exhibiting tetra‐allelic editing, defined by complete loss of the *Nco*I restriction site across all four *NbPDS* alleles as determined by PCR‐RE analysis. RT‐PCR analysis was used to detect residual viral RNA, with Actin as an internal control. WT/U and WT denote wild‐type plants without or with restriction digestion, respectively. (c) Representative regenerated M0 plants (lines M0‐2 and M0‐4) displaying a complete albino phenotype. (d) Indel profiles of selected M0 lines determined by Sanger sequencing and ICE analysis, including examples of tetra‐allelic knockout mutations (line M0‐3) and in‐frame indels that retain partial gene function of NbPDS (line M0‐13). Segregation analysis of M1 progeny from line M0‐13 revealed stable inheritance of the induced mutations. Scale bar = 1.0 cm.

To determine whether *in cis*‐CymMV‐mediated VIGE could generate transgene‐free plants carrying edited alleles transmissible to progeny through regeneration of somatically edited tissues, leaf explants were collected at 6 dpi from *N. benthamiana* leaves infiltrated with *Agrobacterium* harboring pKCyCas9‐NbPDSgRNA. The explants were regenerated on shoot induction medium containing timentin to suppress residual *Agrobacterium*, but without plant‐selective antibiotics. After approximately 1 month of cultivation, regenerated shoots with albino phenotypes were observed (Figure 3a), indicating a high level of somatic editing in *NbPDS*.

A total of 21 independent M0 lines were subjected to molecular characterization. PCR‐RE analysis revealed that 17 of 21 lines (81%) carried edits at the *NbPDS* locus (Figure 3b, lines 1–7, 10, 12–17, 20, and 22–23). Among these edited lines, 14 of 17 (82%) were free of Cas9 transgene and CymMV RNA, as assessed by genomic PCR and RT‐PCR (Figure 3b, lines 1–7, 12–13, 15, 17, 20, and 22–23). Notably, 8 of 21 lines (38%) exhibited complete digestion resistance, indicating complete editing at the *PDS* locus (Figure 3b, lines 1–7 and 13). Consistent with this, representative M0 lines (M0‐2 and M0‐4) displayed a complete albino phenotype (Figure 3c). Sequencing of the amplicon flanking the target site, followed by ICE analysis of selected lines, revealed diverse indel profiles. Line M0‐3 showed a predominance of frameshift mutations (>95%) (Figure 3d). Interestingly, although line M0‐13 had a digestion‐resistant amplicon product (Figure 3b), the plant was normal and showed no phenotype. Sequence analysis indicated 97% editing with a high proportion of in‐frame indels (44%) in line M0‐13 (Figure 3d). The in‐frame mutation in line M0‐13 corresponded to a single amino acid deletion at Ser49 of NbPDS, indicating that this single‐residue deletion likely preserved partial NbPDS function.

To evaluate heritability, M1 progeny derived from self‐pollinated M0‐13 were analyzed. Seeds obtained from self‐pollinated M0‐13 plant segregated into albino and normal seedlings (Normal:Albino = 29:15; Figure 3d, bottom), indicating transmission of edited *NbPDS* alleles to the next generation. The observed phenotypic distribution is consistent with segregation of functional and non‐functional edited alleles originating from the mosaic line M0‐13 background. Together, these results demonstrate that CymMV‐mediated VIGE enables high‐frequency genome editing, supports recovery of transgene‐free and virus‐free M0 plants, and produces heritable mutations, including tetra‐allelic knockouts, in *N. benthamiana*.

### Ruby‐based counterselection enables efficient identification of transgene‐free edited plants

**Figure 4:**
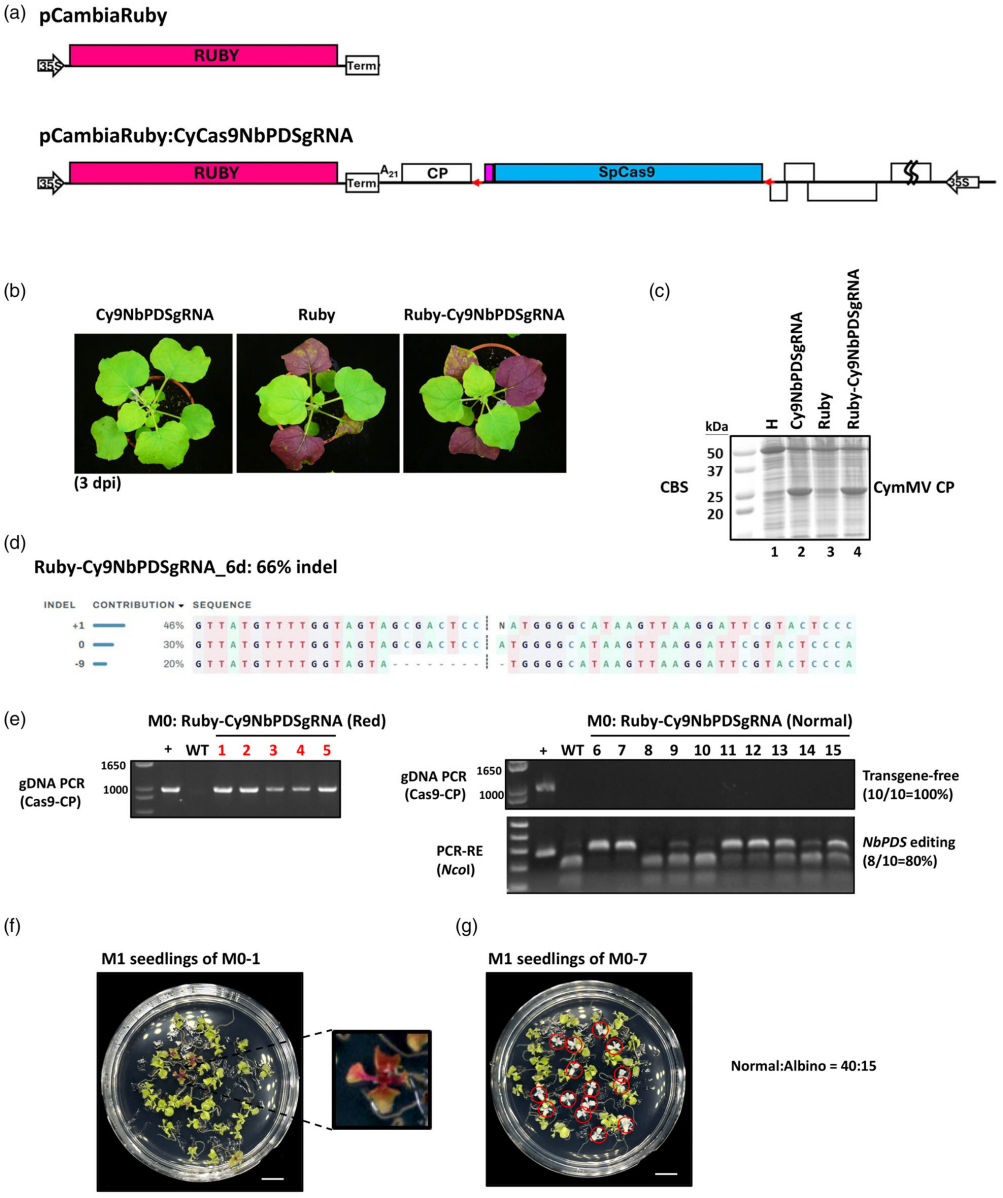
Development of a Ruby‐based counterselection system. (a) Schematic of the dual‐function vector (pCambiaRuby:CyCas9NbPDSgRNA) containing the Ruby reporter gene cassette for visual counterselection. (b) Visible betalain pigmentation in *Nicotiana benthamiana* leaves at 3 dpi, indicating Ruby expression. (c) Coomassie Blue‐stained (CBS)‐stained SDS‐PAGE showing that RUBY expression does not affect cymbidium mosaic virus (CymMV) accumulation at 6 dpi. (d) Indel analysis of *NbPDS* editing efficiency in Ruby‐containing vectors. (e) Molecular analysis of regenerated M0 lines derived from the Ruby‐containing CymMV vector. Genomic DNA PCR was used to detect Cas9‐coat protein (CP) sequences in red M0 regenerants (lines 1–5) and normal‐colored M0 regenerants (lines 6–15). PCR‐RE analysis with *Nco*I was used to examine *NbPDS* target‐site mutations in normal‐colored M0 regenerants. (f) M1 seedlings derived from the red M0 line M0‐1. The enlarged inset shows a representative red‐pigmented M1 seedling. (g) M1 seedlings derived from the normal‐colored, transgene‐free edited M0 line M0‐7. Red circles indicate albino seedlings. Scale bar = 1 cm.

To efficiently identify transgene‐free edited plants after CymMV‐mediated VIGE, we incorporated a Ruby‐based visual counterselection module into the CymMV CRISPR‐Cas9 vector (Figure 4a, lower panel). The Ruby cassette encodes three enzymes of a biosynthetic pathway that convert tyrosine into red betalain pigments, enabling direct visual discrimination of transgene‐containing tissues (He et al., 2020). Ruby expression was driven by the CaMV 35S promoter in the orientation opposite the viral genome (Figure 4a), enabling its use as a negative selectable marker during plant regeneration.

After agroinfiltration of the construct with RUBY and CymMV, strong red pigmentation was readily observed in *N. benthamiana* leaves at 3 dpi (Figure 4b, right panel). Leaves infiltrated with the Ruby‐only control (pCambiaRuby) showed comparable pigmentation intensity (Figure 4b, compare middle and right panels), indicating that viral infection does not interfere with Ruby‐mediated betalain accumulation.

Protein analyses indicated that inclusion of the Ruby cassette did not affect viral accumulation relative to the corresponding non‐Ruby CymMV construct (Figure 4c, compare lanes 2 and 4). Consistent with this, sequencing and ICE analyses revealed that *NbPDS* editing efficiencies mediated by the Ruby‐containing CymMV vector reached approximately 66% (Figure 4d) in infiltrated leaves, comparable to those obtained with the non‐Ruby CymMV vector (53%; Figure 1f). These results indicate that the Ruby counterselection module does not compromise CymMV‐mediated VIGE performance.

Leaf explants infected with the Ruby‐containing CymMV vector were regenerated without antibiotic selection. Regenerated M0 shoots were classified according to the presence or absence of Ruby‐associated red pigmentation and were subsequently subjected to molecular analysis. Genomic DNA PCR showed that red M0 regenerants retained Cas9‐CP transgene sequences, whereas all tested normal‐colored M0 regenerants were free of detectable Cas9‐CP transgene sequences (Figure 4e). PCR‐RE analysis further showed that 80% of these transgene‐free normal‐colored M0 lines carried detectable *NbPDS* edits (Figure 4e, lines 6–7, 9, and 11–15). These results support the use of Ruby pigmentation as a visual counterselection marker for enriching transgene‐free edited plants.

Furthermore, M1 progeny derived from the red M0 line M0‐1 retained Ruby‐associated red pigmentation, consistent with inheritance of the transgenic Ruby cassette (Figure 4f). Separately, M1 progeny derived from the normal‐colored, transgene‐free edited M0 line M0‐7 did not show Ruby‐associated pigmentation but segregated into normal and albino seedlings (Normal:Albino = 40:15), confirming stable transmission of *NbPDS* mutations in a transgene‐free background (Figure 4g). Collectively, these results demonstrate that Ruby‐based counterselection is a robust and efficient strategy for enriching transgene‐free edited plants, substantially simplifying downstream screening in CymMV‐mediated VIGE workflows.

### CymMV‐mediated genome editing in *Phalaenopsis aphrodite*

To evaluate the applicability of the CymMV‐based VIGE platform in orchids, we next applied the system to *P. aphrodite*, a species in which stable genetic transformation remains relatively inefficient and which exhibits a prolonged vegetative growth period (Iiyama et al., 2024; Tiwari et al., 2024). Previous results showed that expression of the *Odontoglossum* ringspot virus (ORSV) P126 protein, an RNA silencing suppressor, is sufficient to enhance CymMV accumulation by promoting cell‐to‐cell movement in *N. benthamiana* (Lee et al., 2021). We therefore evaluated the effect of P126 on CymMV expression in orchid leaves. Consistent with this, co‐infiltration of CymMV‐GFP with P126 resulted in markedly enhanced and sustained GFP fluorescence in orchid tissues at both 10 and 20 dpi, compared with CymMV‐GFP alone (Figure S4). GFP fluorescence was also detected in non‐inoculated upper leaves in the presence of P126, as indicated by white arrows in Figure S4, suggesting that P126 enhanced CymMV‐GFP accumulation and promoted viral spread into newly developed orchid tissues.

**Figure 5:**
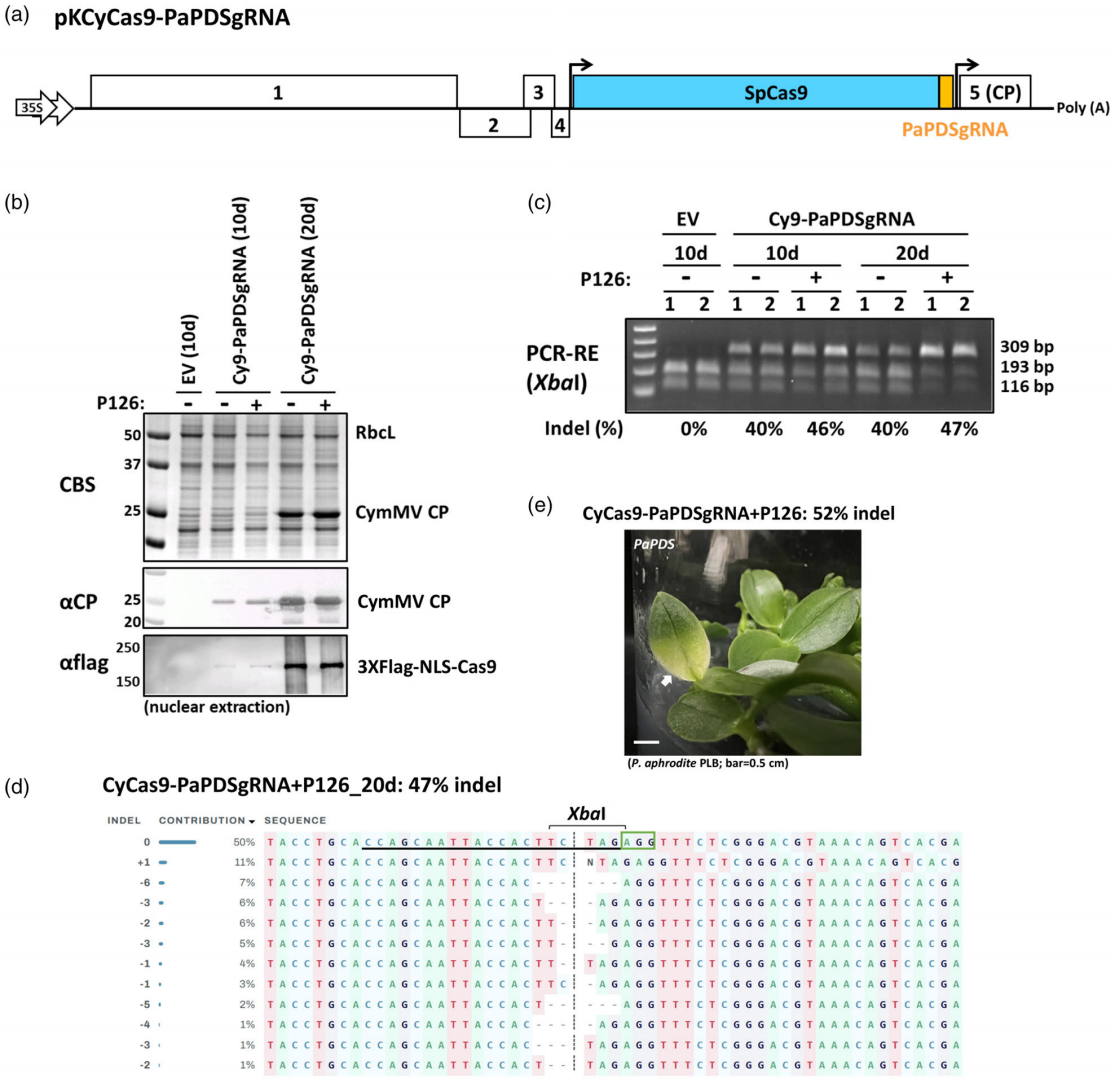
Cymbidium mosaic virus (CymMV)‐mediated virus‐induced genome editing (VIGE) in *Phalaenopsis aphrodite*. (a) Schematic of the CymMV vector construct targeting the *P. aphrodite PDS* gene (pKCyCas9‐PaPDSgRNA). (b) Western blot analysis of Cas9 (α‐Flag) and viral coat protein (CP) expression in orchid leaves co‐infiltrated with or without the P126 silencing suppressor at 10 and 20 dpi. (c) PCR‐RE (*Xba*I) assays detecting indels in the *PaPDS* gene. (d) ICE analysis of Sanger sequencing data showing the distribution of indel patterns. (e) Phenotypes of *P. aphrodite* protocorm‐like bodies (PLBs) inoculated with the editing vector. White arrows indicate localized bleaching phenotypes in infected tissues. Scale bar = 0.5 cm.

Given this improvement in viral accumulation, we next tested whether P126‐assisted CymMV delivery could enhance genome editing efficiency in orchids. A CymMV vector expressing Cas9 and a gRNA targeting the *P. aphrodite PDS* (*PaPDS*) gene was constructed (Figure 5a; Figure S5) and introduced into *P. aphrodite* leaves by *Agrobacterium*‐mediated agroinfiltration, with or without P126 co‐expression. Immunoblot analyses confirmed accumulation of viral CP and nuclear‐localized Cas9 in orchid leaves, with substantial protein accumulation levels at 20 dpi (Figure 5b). Genome editing activity at the *PaPDS* locus was evaluated using PCR‐RE assays targeting an *XbaI* restriction site overlapping the *PaPDS* gRNA1 target (Figure S5). In the absence of P126, indel frequencies of approximately 40% were detected at both 10 and 20 dpi, whereas co‐expression with P126 further increased editing efficiencies to approximately 46–47%, with the highest indel frequency observed at 20 dpi (Figure 5c). Sequence analysis of the edited *PaPDS* locus revealed diverse indel patterns confirming CRISPR‐Cas9 mediated editing in orchid tissues (Figure 5d). Although targeted editing was readily detected in agroinfiltrated mature orchid leaves, visible PDS‐associated bleaching was difficult to observe in these highly differentiated tissues. Therefore, as a separate experiment, we inoculated independently generated *P. aphrodite* protocorm‐like bodies (PLBs) with the CymMV editing vector, pKCyCas9‐PaPDSgRNA. PLBs were used because they continuously produce newly developing tissues, thereby providing an opportunity to monitor visible phenotypes arising from somatic *PaPDS* disruption. PLBs inoculated with the CymMV editing vector exhibited localized bleaching in newly developed leaf tissues at approximately 180 dpi (Figure 5e, indicated by white arrow). ICE analysis of the *PaPDS* target site in newly developed tissues from CymMV‐inoculated PLBs revealed a 52% indel frequency, providing molecular support for somatic editing in PLB tissues (Figure 5e).

Sequence alignment analysis revealed that the *PaPDS* gRNA1 target site is identical among multiple *Phalaenopsis* species (Figure S6a). Consistent with this conservation, application of the identical CymMV‐based Cas9‐PaPDSgRNA construct in *Phalaenopsis equestris* resulted in efficient genome editing, achieving an indel frequency of approximately 58% at 20 dpi in the presence of P126 (Figure S6b). These results indicate that the *PaPDS*‐targeting CymMV‐VIGE system is readily transferable across *Phalaenopsis* species without the need for species‐specific gRNA redesign.

Together, these results demonstrate that CymMV‐mediated VIGE, when combined with transient suppression of RNA silencing, enables efficient somatic genome editing in *Phalaenopsis* tissues. These data establish a proof‐of‐concept for CymMV‐mediated CRISPR‐Cas9 delivery and rapid gRNA activity validation in orchids.

### Multiplex genome editing in orchids using CymMV‐based VIGE

**Figure 6:**
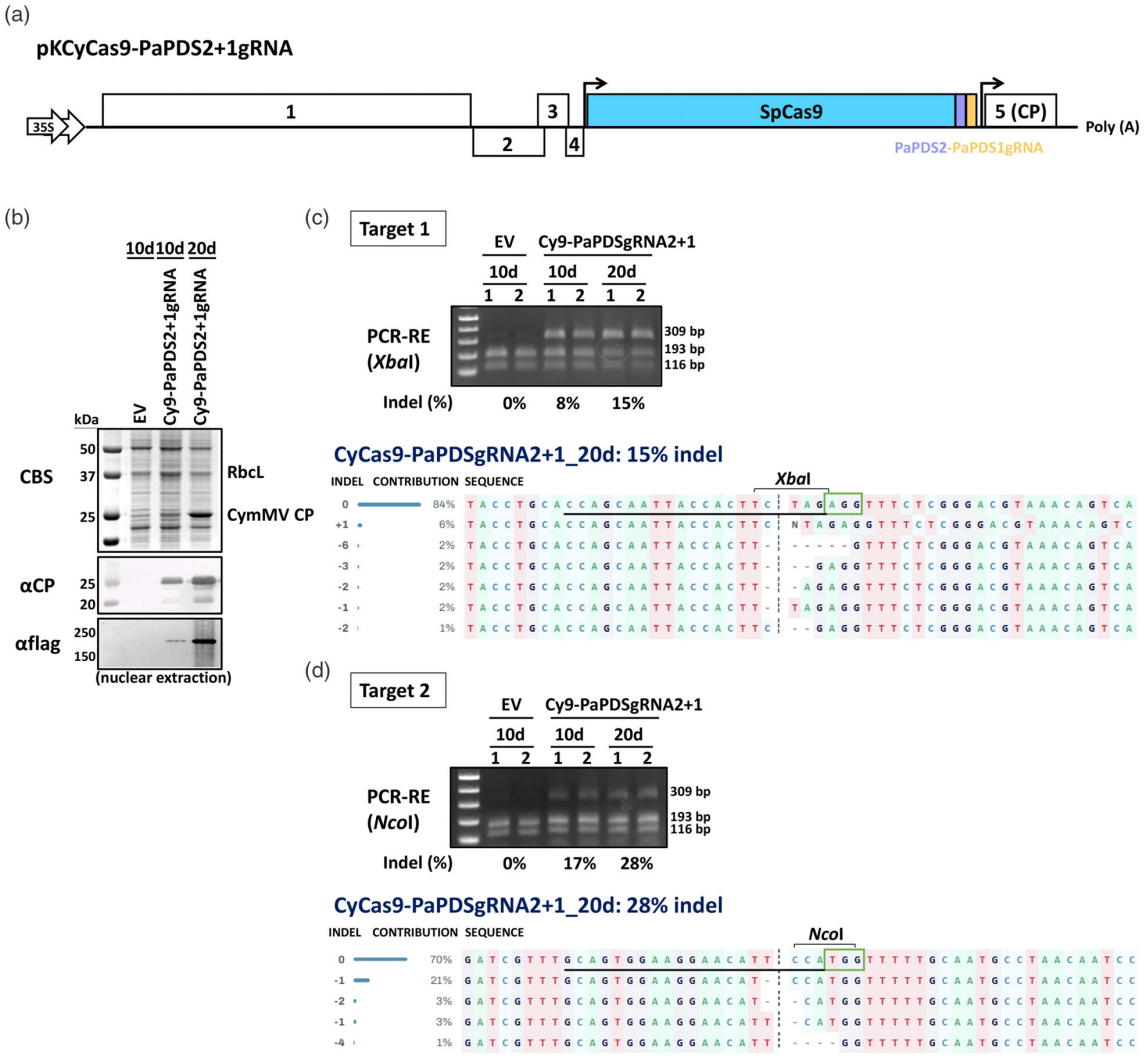
Multiplex genome editing in orchids using a single cymbidium mosaic virus (CymMV) vector. (a) Schematic of the multiplexing vector pKCyCas9‐PaPDS2+1gRNA, designed to express Cas9 and two distinct gRNAs (PaPDS1 and PaPDS2) targeting the *PaPDS* gene. (b) Immunoblot analysis confirming Cas9 and viral coat protein (CP) expression in orchid tissues. (c, d) PCR‐RE assays for the simultaneous detection of mutations at Target 1 (digested with *Xba*I) and Target 2 (digested with *Nco*I). Sequence analysis of the edited loci showing the indel patterns at both target sites.

To examine whether the CymMV‐based VIGE platform can support multiplex genome editing in orchids, we engineered a single CymMV vector expressing Cas9 together with two gRNAs targeting distinct sites within the *PaPDS* gene (Figure 6a). The two gRNAs (PaPDS gRNA1 and PaPDS gRNA2), targeting distinct regions of the *PaPDS* gene and associated with *XbaI* and *NcoI* restriction sites, respectively, were arranged in tandem within the same viral genome to enable simultaneous delivery and expression from a single replicating RNA (Figure S5).

Following agroinfiltration, accumulation of viral CP and nuclear‐localized Cas9 was readily detected at both 10 and 20 dpi, indicating stable expression of the multiplex editing construct in orchid tissues (Figure 6b). Genome editing activity at each target site was independently evaluated using PCR‐RE assays. At 20 dpi, indel frequencies of approximately 15 and 28% were detected at *PaPDS* Target 1 and *PaPDS* Target 2, respectively, whereas no editing was detected in empty vector controls (Figure 6c,d).

Sequence analysis further confirmed the presence of diverse indel patterns at both target sites. Although the editing efficiencies at individual targets were lower than that achieved using a single gRNA construct (Figure 5c), concurrent mutagenesis at two independent genomic sites within the same tissue demonstrates that a single CymMV replicon can stably deliver and express multiple gRNAs in orchids. The reduction in editing efficiency may reflect constraints in gRNA maturation when expressed as tandem arrays without engineered processing elements.

Together, these results indicate that CymMV‐based VIGE is compatible with multiplex genome editing in orchid species and provides a flexible framework for simultaneous manipulation of multiple genomic sites, thereby extending the utility of this platform toward more complex trait engineering applications.

## DISCUSSION

**Figure 7:**
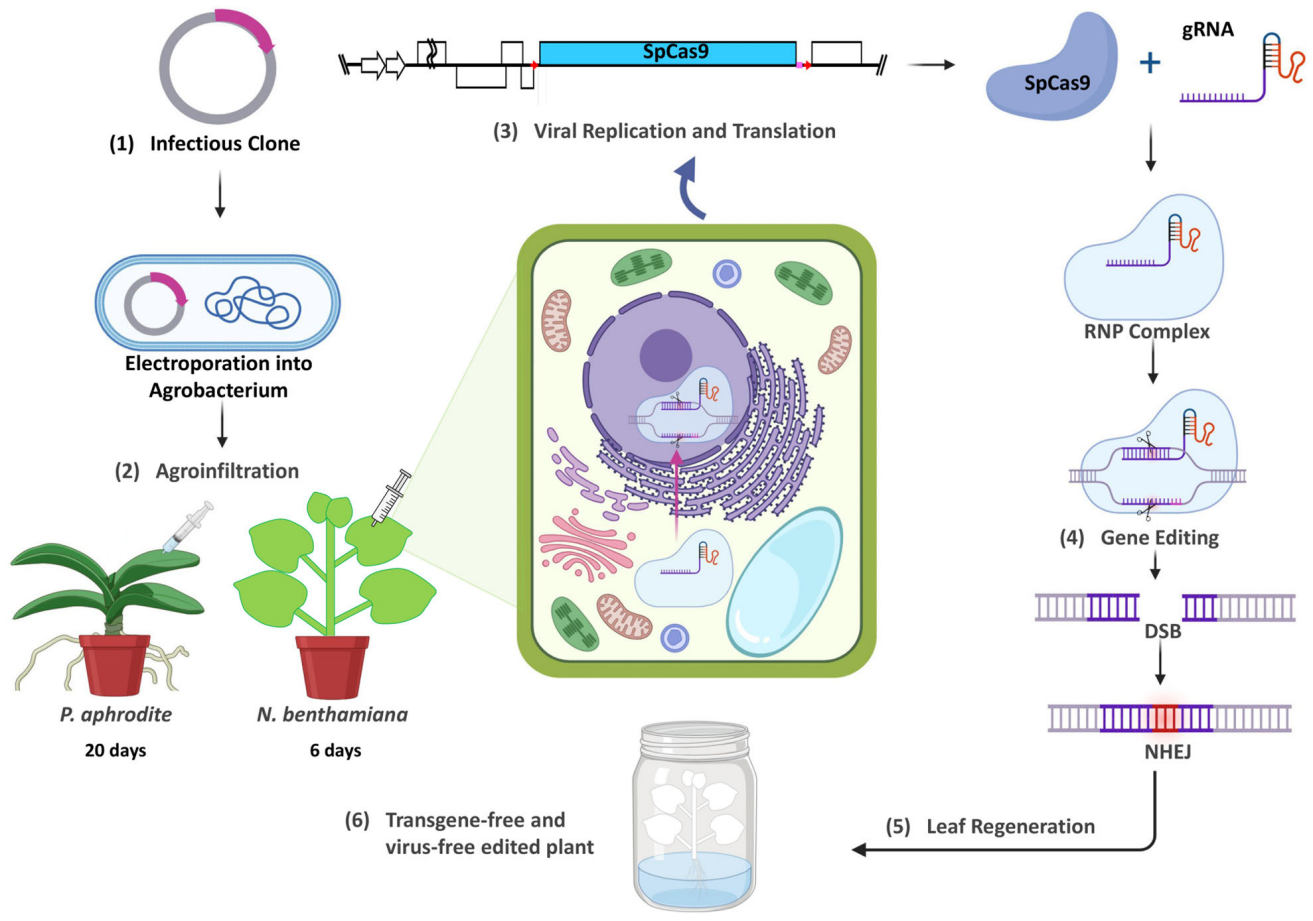
Workflow of cymbidium mosaic virus (CymMV)‐mediated transgene‐free genome editing in plants. Schematic representation of the CymMV‐based VIGE platform. (1) An infectious CymMV clone encoding SpCas9 and gRNA is introduced into *Agrobacterium* by electroporation. (2) Agroinfiltration delivers the viral cDNA into tissues of *Nicotiana benthamiana* or *Phalaenopsis aphrodite*. (3) Following viral replication in plant cells, subgenomic RNA transcription drives SpCas9 expression, while gRNA is concurrently produced from the viral replicon. (4) Cas9 associates with gRNA to form a ribonucleoprotein (RNP) complex that is transported into the nucleus, where it induces a double‐strand break (DSB) at the target locus. DNA repair via non‐homologous end joining (NHEJ) results in indel mutations. (5) Edited tissues are regenerated. (6) Regenerated plants can be recovered as transgene‐free and virus‐free genome‐edited lines. Detectable somatic editing occurs approximately 6 days post‐inoculation in *N. benthamiana* and 20 days in *P. aphrodite*.

In this study, we established a CymMV‐mediated VIGE platform that enables efficient, rapid, and transgene‐free genome editing in plants. As summarized in the proposed workflow (Figure 7), the system uses a simplified vector design that enables concurrent expression of Cas9 and gRNA from a single CymMV replicon, thereby streamlining the genome‐editing process. After agro‐infiltration, somatic editing can be detected at as early as 6 days in *N. benthamiana* and 20 days in *Phalaenopsis* species (Figures 1 and 5; Figure S6). This short time frame also facilitates rapid *in planta* evaluation of gRNA activity prior to stable regeneration, which is particularly valuable in orchids, where transformation and phenotypic validation are typically prolonged.

A key advantage of this workflow is the high editing efficiency during the initial infection phase, enabling recovery of transgene‐free, fully edited alleles, including tetra‐allelic knockout events, already in the M0 generation of *N. benthamiana* (Figure 3). In addition, the CymMV‐based VIGE platform supports multiplex genome editing by simultaneously expressing multiple gRNAs from a single viral replicon, allowing concurrent modification of multiple target sites without additional vector complexity (Figure 6). Moreover, the incorporation of a Ruby‐based visual counterselection module further facilitates the direct identification of transgene‐free edited lines. Specifically, regenerated plants exhibiting a normal green phenotype, rather than Ruby‐associated red pigmentation, were selected, thereby enriching for genome‐edited individuals free of transgene sequences (Figure 4). Importantly, regenerated plants were free of detectable viral RNA (Figure 3b), suggesting that genome editing occurs during transient CymMV replication and is effectively uncoupled from sustained viral infection during regeneration, thereby avoiding concerns about viral contamination.

The markedly higher editing efficiency observed with the *in cis* configuration compared with the *in trans* strategy may reflect fundamental differences in how CRISPR components are delivered and amplified within infected cells. In the *in cis* design, both Cas9 and the gRNA are encoded on a single CymMV replicon, allowing their expression to be tightly coupled to viral replication. This coordinated amplification ensures that both components accumulate concurrently and at high levels in the same infected cells, thereby facilitating efficient RNP assembly and genome cleavage (Cody & Scholthof, 2020). In contrast, the *in trans* strategy relies on gRNA expression from a non‐replicating T‐DNA driven by a Pol III promoter, whereas Cas9 expression benefits from viral replication. This separation may result in differences in the spatial or temporal coordination of Cas9 and gRNA expression, which could contribute to reduced editing efficiency relative to the *in cis* configuration. This interpretation is further supported by the non‐viral transient expression assay, in which the *in cis* configuration consistently yielded higher indel frequencies than the corresponding *in trans* design. Our findings align with studies using other potexvirus‐based vectors, such as potato virus X (PVX) and bamboo mosaic virus (BaMV), in which coupling genome editing components on a single replicating vector was critical for achieving robust systemic editing (Ariga et al., 2020; Wu et al., 2025). Therefore, the *in cis* strategy effectively exploits the viral amplification machinery to overcome the dosage bottlenecks commonly encountered in transient delivery systems. In addition to coordinated viral amplification, the generation of functional gRNAs from longer viral‐derived transcripts may also contribute to the efficiency of the *in cis* design. In the *in cis* CymMV design, the gRNA is expressed as part of a longer viral‐derived transcript rather than expressed as a canonical Pol III transcript. Accumulating evidence from RNA virus‐based systems indicates that gRNAs present within longer transcripts can remain functional *in planta*. Similar observations have been reported for tobacco mosaic virus‐derived TRBO vectors and PVX‐based platforms, in which gRNAs containing substantial 5′ leader sequences and 3′ untranslated regions nevertheless supported efficient genome editing (Cody et al., 2017; Uranga et al., 2021). This phenomenon has been attributed to native RNA processing and/or Cas9‐assisted maturation, whereby Cas9 binding stabilizes the gRNA core while endogenous ribonucleases trim unprotected flanking sequences (Cody & Scholthof, 2020; Mikami et al., 2017). Consistent with this model, our fractionation RT‐PCR analysis showed that upstream Cas9‐linked viral RNA regions were detected in the total RNA fraction but not in the nuclear‐enriched fraction (Figure S2b), suggesting that the gRNA present in the nuclear fraction may represent processed Cas9‐gRNA complexes rather than intact viral transcripts. This mechanism may operate in the CymMV system, allowing functional Cas9‐gRNA complexes to form from viral‐derived transcripts without engineered processing elements such as ribozymes or tRNA spacers. However, in the multiplex construct, multiple gRNAs arranged in tandem may be processed or matured less efficiently, which could contribute to the reduced editing efficiency observed compared with the single‐gRNA construct (Figure 6).

A notable feature of the CymMV‐mediated VIGE system developed in this study is the frequent recovery of virus‐free edited plants directly from the M0 generation (Figure 3), without antiviral selection or subsequent genetic segregation. This behavior contrasts with several previously reported viral genome editing platforms in *N. benthamiana*. For example, PVX‐based vectors, despite belonging to the same *Potexvirus* genus, commonly result in persistent viral infection in regenerated shoots, reflecting the high replication capacity and efficient systemic movement of PVX in its natural solanaceous hosts (Ariga et al., 2020). Similarly, plants regenerated from tissues infected with sonchus yellow net rhabdovirus (SYNV) often retain viral RNA, necessitating an additional generational step to obtain virus‐free progeny (Ma et al., 2020, 2023). The ability to recover virus‐free plants following regeneration in the CymMV system may be attributed to reduced compatibility between CymMV and the heterologous host *N. benthamiana*. In this host, CymMV does not readily establish systemic infection (Lee et al., 2021), and its cell‐to‐cell movement is likely less efficient than that of highly adapted potexviruses such as PVX (Beernink et al., 2022). During regeneration, rapid cellular proliferation associated with shoot formation may outpace viral spread, diluting and eventually eliminating the virus from regenerating tissues. As a result, genome editing occurs during a transient phase of CymMV replication, while regenerated plants are free of detectable viral RNA (Figure 3b). This feature represents a practical advantage of the CymMV‐based VIGE platform, as it eliminates the need for additional generations or antiviral treatments to remove viral infection (Liu et al., 2023), thereby streamlining genome editing workflows—particularly for vegetatively propagated crops or species with long generation times, where seed‐based virus clearance is impractical. Alternatively, genome editing can be achieved using a CP‐deleted CymMV replicon, which retains substantial somatic editing activity while restricting viral movement (Figure 2). The moderate reduction in editing efficiency observed with the CP‐deleted replicon likely reflects impaired viral spread rather than insufficient Cas9 accumulation, because this construct produced higher levels of nuclear‐localized Cas9 than the full‐length vector (Figure 2b). This observation suggests that Cas9 abundance alone is unlikely to be the primary limiting factor under these conditions. Restricted cell‐to‐cell movement may reduce the number of cells receiving both Cas9 and gRNA, thereby lowering the overall editing frequency in infiltrated tissues. Thus, the CP‐deleted CymMV replicon represents a trade‐off between editing efficiency and viral containment. This design provides an additional layer of biocontainment by minimizing viral spread and may provide a practical and controllable platform for localized plant genome editing applications.

*Phalaenopsis* orchids, a major sector of the global floriculture market, have lacked a compatible viral vector capable of delivering the full CRISPR editing payload. CRISPR/Cas9‐mediated genome editing has been demonstrated in *Phalaenopsis* and *Dendrobium* using conventional *Agrobacterium*‐based transformation systems (Iiyama et al., 2024; Kui et al., 2017). Stable integration of Cas9 and gRNA expression cassettes has enabled targeted mutagenesis of floral MADS‐box genes and the *PDS* locus in T0 transformants after antibiotic selection (Semiarti et al., 2020; Tong et al., 2019), demonstrating the functionality of CRISPR/Cas9 in orchid cells. However, these approaches generally involve genomic integration of editing constructs. Because orchids are typically propagated vegetatively and exhibit long juvenile phases, segregation‐based removal of transgenes can be challenging or time‐consuming. In addition, transformation and regeneration efficiencies can be genotype‐dependent and may also vary with experimental protocols, which may affect the broader application of stable transformation‐based editing approaches (Iiyama et al., 2024; Tiwari et al., 2024).

The CymMV‐mediated VIGE system described here is designed to deliver CRISPR‐Cas9 components through transient viral replication in orchid tissues. By delivering Cas9 and gRNAs from a single replicating RNA molecule, this approach enables effective somatic genome modification in orchid tissues while avoiding permanent genomic insertion. In *Phalaenopsis*, the system supported efficient editing in infiltrated leaves, providing a rapid platform for evaluating gRNA activity directly in orchid tissues. Although the present orchid experiments demonstrate somatic editing rather than recovery of fully regenerated edited plants, they establish an important foundation for CymMV‐mediated genome editing in orchids. A key next step must be the regeneration of edited orchid plants from infected tissues. In this regard, PLBs represent a particularly suitable target because they are highly regenerative, clonally propagated tissues and are widely used in orchid transformation workflows (Iiyama et al., 2024). In this study, we confirmed that CymMV‐based editing vectors can infect PLBs and induce localized PaPDS‐associated bleaching in newly developed tissues (Figure 5e), suggesting that CymMV‐infected PLBs may serve as suitable regenerative starting materials for future recovery of edited orchid plants. Building on this result, future work should focus on optimizing regeneration from edited PLBs and establishing molecular screening workflows to recover edited, transgene‐free, and virus‐free orchid plants. Although further investigation is needed to determine the extent of viral clearance during orchid regeneration, the recovery of virus‐free edited plants in *N. benthamiana* indicates that viral elimination may be possible under certain conditions, warranting careful evaluation in orchid hosts. Building on this framework, the recent emergence of hypercompact RNA‐guided nucleases, including AsCas12f and TnpB, which have been deployed in TRV‐, PVX‐, and BaMV‐based systems (Hu et al., 2026; Ishibashi et al., 2024; Nagalakshmi et al., 2026; Weiss et al., 2025; Wu et al., 2025), offers additional opportunities to further refine CymMV‐based editing approaches; beyond their reduced coding size, these nucleases recognize distinct PAM sequences relative to SpCas9, which may broaden the range of editable target sites within the complex orchid genomes. In addition to its experimental utility, the CymMV‐based VIGE system may also have practical relevance for the commercial orchid industry. CymMV is widely distributed and naturally infects multiple economically important orchid species, including *Phalaenopsis*, *Dendrobium*, *Oncidium*, *Epidendrum*, *Laelia*, *Vanda*, and *Zygopetalum* (Yusop et al., 2022). This broad host compatibility suggests that a CymMV‐based editing platform could be adaptable across diverse orchid species. Collectively, establishing a CymMV‐based VIGE system expands the current genome editing toolkit for orchids and provides a species‐adapted approach that may facilitate future functional genomics and precision breeding efforts in this important ornamental crop.

## MATERIALS AND METHODS

### Plant materials and growth conditions

*Nicotiana benthamiana* plants were grown in a greenhouse under controlled conditions at 28°C with a 16 h light/8 h dark photoperiod.

*Phalaenopsis aphrodite* plants at the four‐leaf stage were obtained from CLONE International Biotech Co. (Pingtung, Taiwan) and maintained in a greenhouse at 25°C under a 12 h light/12 h dark photoperiod.

### Construction of CymMV‐based genome editing vectors

All primers used for the plasmid construction are listed in Table S1. The CymMV‐based genome editing vectors were constructed using a previously described CymMV infectious clone as the backbone (Lee et al., 2021). To generate a Cas9‐expressing CymMV vector (pKCyCas9), the full‐length *S. pyogenes* Cas9 (SpCas9) coding sequence was digested from pKSE401 (Xing et al., 2014) using *Xba*I and *Sac*I, converted to blunt ends with T4 DNA polymerase, and ligated into the *Not*I‐digested, blunt‐ended pKCy1GFP vector (Lee et al., 2021). For construction of the CP‐deleted Cas9 vector, the 3′ untranslated region (3′ UTR) of CymMV was amplified by PCR using the primer pair PacI/Cy3′UTR_F and SacI/oligodT21 (Table S1), gel‐purified, digested with *Pac*I and *Sac*I, and subsequently cloned into pKCyCas9 digested with the same enzymes to generate pKCydCCas9.

Single‐gRNAs targeting *NbPDS* or *PaPDS* were designed using the CRISPRdirect web tool (Naito et al., 2015). Cassettes for gRNA expression were amplified by PCR from the pKSE401 template using the corresponding primers listed in Table S1, digested with *Pac*I, and cloned into pKCyCas9 and pKCydCCas9 to generate CymMV vectors carrying individual gRNAs, pKCyCas9‐NbPDSgRNA, pKCydCCas9‐NbPDSgRNA, and pKCyCas9‐PaPDSgRNA, respectively. For multiplex genome editing, an additional PaPDS2 gRNA cassette was sequentially inserted into pKCyCas9‐PaPDSgRNA, generating pKCyCas9‐PaPDS2+1 capable of expressing Cas9 and two gRNAs from a single viral genome.

To construct the plasmid pCambiaRuby, the 35S:RUBY expression cassette was digested from Addgene plasmid #160908 (He et al., 2020) using *Spe*I and *Hind*III, followed by blunt‐end conversion with T4 DNA polymerase, and cloned into the *Nco*I/*Pml*I‐digested, blunt‐ended pCambia1305.1 backbone.

The CyCas9‐NbPDSgRNA cassette was subsequently digested from pKCyCas9‐NbPDSgRNA using *Sbf*I and *Sac*I and cloned into the pCambiaRuby backbone digested with the same enzymes to generate pCambiaRuby:CyCas9NbPDSgRNA.

### Plant inoculation by agroinfiltration

CymMV‐based constructs used for agroinfiltration in *N. benthamiana* were introduced into *Agrobacterium tumefaciens* strain GV3850 by electroporation. Transformed cultures were grown at 28°C, collected by centrifugation, and resuspended in infiltration buffer (10 mM MgCl_2_, 10 mM MES, pH 5.5, and 200 μM acetosyringone [AS]). Bacterial suspensions were adjusted to an OD_600_ of 0.5 and infiltrated into leaves using a needleless syringe.

For agroinfiltration of *P. aphrodite*, CymMV constructs were electroporated into *A. tumefaciens* strain EHA105. Bacterial cells were collected by centrifugation and resuspended in AB‐MES +1/2 MS infiltration buffer containing 200 μM AS, as described previously (Kuo et al., 2021). After adjustment to an OD_600_ of 0.5, the suspensions were infiltrated into orchid leaves using a needleless syringe. Where indicated, *Agrobacterium* carrying the ORSV P126 construct was co‐infiltrated to enhance viral accumulation.

### Generation and *Agrobacterium*‐mediated inoculation of *Phalaenopsis* PLBs

Mature seeds of *P. aphrodite* were sown on half‐strength modified Murashige and Skoog medium (½MS) containing half‐strength macro‐ and micro‐elements, supplemented with 1000 mg L^−1^ peptone, 20 000 mg L^−1^ sucrose, and 7000 mg L^−1^ agar. Cultures were incubated at 26°C under a 12‐h photoperiod. After 90 days of culture, seeds germinated into zygotic protocorms approximately 0.5 mm in diameter. The protocorms were then bisected and transferred onto the same ½MS medium supplemented with 2 mg L^−1^ 6‐benzylaminopurine (BA) for PLB induction.

For PLB inoculation, PLBs of *P. aphrodite* were pre‐cultured for 24 h on a modified seed germination medium (SD medium; Hu et al., 2013) supplemented with 1% (w/v) activated charcoal and 100 μM AS. *Agrobacterium tumefaciens* strain EHA105 harboring the pKCyCas9‐PaPDSgRNA vector was resuspended in liquid SD medium containing 100 μM AS to an OD_600_ of 1.0. The pre‐cultured PLBs were immersed in this bacterial suspension, sonicated for 3 min, and subsequently co‐cultivated on solid SD medium with AS for 3 days. Following co‐cultivation, the PLBs were thoroughly washed with liquid SD medium containing 200 mg L^−1^ timentin and transferred onto fresh solid SD medium supplemented with 200 mg L^−1^ timentin to suppress bacterial growth.

### Protein analysis

Protein extraction and nuclear fractionation were performed essentially as described by Xu and Copeland (2012), with minor modifications. Briefly, agroinfiltrated tissues were ground in liquid nitrogen and homogenized in lysis buffer. Total protein samples were collected from the crude homogenates prior to centrifugation during the nuclear extraction procedure, and nuclear fractions were subsequently isolated following the published protocol.

Equal amounts of protein were separated by SDS‐PAGE and subsequently visualized by Coomassie Blue staining (CBS) or analyzed by immunoblotting. For immunoblotting, proteins were transferred onto PVDF membranes (Millipore, Burlington, MA, USA), which were incubated with laboratory‐produced rabbit polyclonal antibodies against CymMV CP or the FLAG epitope (1:5000 dilution), followed by incubation with the corresponding secondary antibodies for signal detection.

### RT‐PCR analysis

Total RNA was isolated from nuclear fractions using a standard phenol–chloroform extraction method and treated with RNase‐free DNase I. Reverse transcription was performed using gene‐specific primers, PDSsgRNA_R for gRNA and U6snRNA_R for U6 small nuclear RNA (snRNA), respectively. PCR amplification was carried out to detect nuclear‐localized gRNA, with U6 snRNA used as an internal control. Primer sequences used for RT‐PCR are listed in Table S1.

For detection of viral RNA in M0 plants, RT was carried out using oligo(dT)_18_ primer, followed by PCR amplification with primers Cas9‐4130F and Cyy1‐6217R (Table S1), which amplify a region spanning Cas9 and the CymMV CP (Cas9‐CP).

### Genome editing analysis

Genomic DNA was extracted from agroinfiltrated leaf tissues or regenerated plant materials using a standard cetyltrimethylammonium bromide‐based method (Allen et al., 2006). Target regions spanning the gRNA binding sites were amplified by PCR using locus‐specific primers (Table S1).

Genome editing was initially assessed by PCR‐restriction enzyme (PCR‐RE) analysis. PCR amplicons were digested with the corresponding restriction enzymes recognizing sites overlapping the gRNA target sequences (*NcoI* or *XbaI*, as indicated) and resolved by agarose gel electrophoresis.

For sequence‐based analysis, PCR products were purified and subjected to Sanger sequencing. Indel frequencies were quantified using ICE software v2.0 (Synthego, Redwood City, CA, USA; Conant et al., 2022) based on Sanger sequencing chromatograms.

### Leaf regeneration from agroinfiltrated *N. benthamiana* leaves

Agroinfiltrated *N. benthamiana* leaves were collected at 6 dpi, surface‐sterilized with 10% (v/v) bleach solution containing 0.05% (v/v) Tween 20 for 15 min, and washed three times with sterile water. Leaves were cut into approximately 1 cm^2^ explants and placed on shoot induction medium consisting of Murashige and Skoog (MS) basal salts supplemented with B5 vitamins, 1 ppm 6‐BA, 0.2 ppm 1‐naphthaleneacetic acid, 0.8% (w/v) agar, and 500 mg L^−1^ timentin. Timentin was used to suppress and eliminate residual *Agrobacterium*. Regenerated shoots were excised and transferred to MS‐based rooting medium to induce root formation.

## AUTHOR CONTRIBUTIONS

Y.‐W.H. designed the methodology, performed the experiments, and wrote the original draft of the manuscript. C.‐C.H. and Y.‐H.H. conceived and designed the research, interpreted the data, and revised the manuscript. Y.‐H.C. performed the orchid protocorm‐like body (PLB) infection experiments. C.‐H.T. and N.‐S.L. provided study materials, participated in data discussion, and reviewed the manuscript. S.P.D.‐K. participated in data discussion and revised the manuscript.

## CONFLICT OF INTEREST

None of the authors have a conflict of interest to disclose.

## Supporting information


**Figure S1.** Schematic representation of the NbPDS gene structure in the allotetraploid *N. benthamiana*, comprising two homoeologs, *NbPDS‐A* and *NbPDS‐B*.
**Figure S2.** RT‐PCR analysis of gRNA‐associated RNA species in total and nuclear‐enriched RNA fractions.
**Figure S3.** The *in cis* Cas9‐gRNA expression strategy enhances transient genome editing efficiency.
**Figure S4.** ORSV P126 enhances CymMV accumulation and systemic expression in orchids.
**Figure S5.** Target site design for multiplex genome editing in *P. aphrodite*.
**Figure S6.** Conservation of the PaPDS gRNA target site among *Phalaenopsis* species and validation of CymMV‐mediated genome editing in *Phalaenopsis equestris*.
**Table S1.** List of primers used in this study.

## Data Availability

Sequence data from this article can be found in the Solanaceae Genomics Network under accession numbers: *NbPDS‐A* (Niben101Scf01283Ctg022), *NbPDS‐B* (Niben101Scf14708Ctg002) and in the Orchidstra 2.0 database: *PaPDS* (PAXXG009450). The data underlying the genome‐editing analyses are available in Zenodo under DOI: 10.5281/zenodo.20669206.

## References

[tpj71031-bib-0001] Ali, Z. , Abul‐Faraj, A. , Li, L. , Ghosh, N. , Piatek, M. , Mahjoub, A. et al. (2015) Efficient virus‐mediated genome editing in plants using the CRISPR/Cas9 system. Molecular Plant, 8, 1288–1291. 25749112 10.1016/j.molp.2015.02.011

[tpj71031-bib-0002] Allen, G.C. , Flores‐Vergara, M.A. , Krasynanski, S. , Kumar, S. & Thompson, W.F. (2006) A modified protocol for rapid DNA isolation from plant tissues using cetyltrimethylammonium bromide. Nature Protocols, 1, 2320–2325. 17406474 10.1038/nprot.2006.384

[tpj71031-bib-0003] Altpeter, F. , Springer, N.M. , Bartley, L.E. , Blechl, A.E. , Brutnell, T.P. , Citovsky, V. et al. (2016) Advancing crop transformation in the era of genome editing. The Plant Cell, 28, 1510–1520. 27335450 10.1105/tpc.16.00196 PMC4981132

[tpj71031-bib-0004] Ariga, H. , Toki, S. & Ishibashi, K. (2020) Potato virus X vector‐mediated DNA‐free genome editing in plants. Plant and Cell Physiology, 61, 1946–1953. 32991731 10.1093/pcp/pcaa123 PMC7758033

[tpj71031-bib-0005] Baysal, C. , Kausch, A.P. , Cody, J.P. , Altpeter, F. & Voytas, D.F. (2025) Rapid and efficient in planta genome editing in sorghum using foxtail mosaic virus‐mediated sgRNA delivery. The Plant Journal, 121, e17196. 39661735 10.1111/tpj.17196 PMC11771572

[tpj71031-bib-0006] Beernink, B.M. , Lappe, R.R. , Bredow, M. & Whitham, S.A. (2022) Impacts of RNA mobility signals on virus induced somatic and germline gene editing. Frontiers in Genome Editing, 4, 925088. 35755451 10.3389/fgeed.2022.925088 PMC9219249

[tpj71031-bib-0007] Chang, H.J. & Ku, H.M. (2024) The current status and prospects of virus‐induced gene editing in plant. Agricultural Research and Technology, 28, 556427.

[tpj71031-bib-0008] Chen, K. , Wang, Y. , Zhang, R. , Zhang, H. & Gao, C. (2019) CRISPR/Cas genome editing and precision plant breeding in agriculture. Annual Review of Plant Biology, 70, 667–697. 10.1146/annurev-arplant-050718-100049 30835493

[tpj71031-bib-0010] Cody, W.B. & Scholthof, H.B. (2020) Native processing of single guide RNA transcripts to create catalytic Cas9/single guide RNA complexes in planta. Plant Physiology, 184, 1194–1206. 32665336 10.1104/pp.20.00150 PMC7536693

[tpj71031-bib-0011] Cody, W.B. , Scholthof, H.B. & Mirkov, T.E. (2017) Multiplexed gene editing and protein overexpression using a tobacco mosaic virus viral vector. Plant Physiology, 175, 23–35. 28663331 10.1104/pp.17.00411 PMC5580747

[tpj71031-bib-0012] Conant, D. , Hsiau, T. , Rossi, N. , Oki, J. , Maures, T. , Waite, K. et al. (2022) Inference of CRISPR edits from sanger trace data. The CRISPR Journal, 5, 123–130. 35119294 10.1089/crispr.2021.0113

[tpj71031-bib-0013] Doudna, J.A. & Charpentier, E. (2014) The new frontier of genome engineering with CRISPRCas9. Science, 346, 1258096. 25430774 10.1126/science.1258096

[tpj71031-bib-0014] Ellison, E.E. , Nagalakshmi, U. , Gamo, M.E. , Huang, P.J. , Dinesh‐Kumar, S. & Voytas, D.F. (2020) Multiplexed heritable gene editing using RNA viruses and mobile single guide RNAs. Nature Plants, 6, 620–624. 32483329 10.1038/s41477-020-0670-y

[tpj71031-bib-0015] Han, X. , Deng, Z. , Liu, H. & Ji, X. (2025) Current advancement and future prospects in simplified transformation‐based plant genome editing. Plants, 14, 889. 40265805 10.3390/plants14060889 PMC11944944

[tpj71031-bib-0016] He, Y. , Zhang, T. , Sun, H. , Zhan, H. & Zhao, Y. (2020) A reporter for noninvasively monitoring gene expression and plant transformation. Horticulture Research, 7, 152. 33024566 10.1038/s41438-020-00390-1 PMC7502077

[tpj71031-bib-0017] He, Y. & Zhao, Y. (2020) Technological breakthroughs in generating transgene‐free and genetically stable CRISPR‐edited plants. aBIOTECH, 1, 88–96. 36305007 10.1007/s42994-019-00013-x PMC9584093

[tpj71031-bib-0018] Hsieh, M.H. , Lu, H.C. , Pan, Z.J. , Yeh, H.H. , Wang, S.S. , Chen, W.H. et al. (2013) Optimizing virus‐induced gene silencing efficiency with cymbidium mosaic virus in *Phalaenopsis* flower. Plant Science, 201, 25–41. 23352400 10.1016/j.plantsci.2012.11.003

[tpj71031-bib-0019] Hu, M. , Zhang, L. , Herrera‐Estrella, L. & Liu, D. (2026) Heritable, tissue culture‐independent and transgene‐free genome editing in plants via viral delivery of CRISPR/AsCas12f. Plant Biotechnology Journal, 24, 84–86. 40974018 10.1111/pbi.70315 PMC12854885

[tpj71031-bib-0020] Hu, W.H. , Yang, Y.H. , Liaw, S.I. & Chang, C. (2013) Cryopreservation the seeds of a Taiwanese terrestrial orchid, *Bletilla formosana* (Hayata) Schltr. by vitrification. Botanical Studies, 54, 33. 28510879 10.1186/1999-3110-54-33 PMC5432767

[tpj71031-bib-0021] Iiyama, C.M. , Vilcherrez‐Atoche, J.A. , Germanà, M.A. , Vendrame, W.A. & Cardoso, J.C. (2024) Breeding of ornamental orchids with focus on *Phalaenopsis*: current approaches, tools, and challenges for this century. Heredity, 132, 163–178. 38302667 10.1038/s41437-024-00671-8 PMC10997592

[tpj71031-bib-0022] Ishibashi, K. , Sukegawa, S. , Endo, M. , Hara, N. , Nureki, O. , Saika, H. et al. (2024) Systemic delivery of engineered compact AsCas12f by a positive‐strand RNA virus vector enables highly efficient targeted mutagenesis in plants. Frontiers in Plant Science, 15, 1454554. 39323536 10.3389/fpls.2024.1454554 PMC11423357

[tpj71031-bib-0023] Jinek, M. , Chylinski, K. , Fonfara, I. , Hauer, M. , Doudna, J.A. & Charpentier, E. (2012) A programmable dual‐RNA‐guided DNA endonuclease in adaptive bacterial immunity. Science, 337, 816–821. 22745249 10.1126/science.1225829 PMC6286148

[tpj71031-bib-0024] Kang, B. , Lee, S. , Ko, D.H. , Venkatesh, J. , Kwon, J.K. , Kim, H. et al. (2025) Virus‐induced systemic and heritable gene editing in pepper (*Capsicum annuum* L.). The Plant Journal, 122, e70257. 40499557 10.1111/tpj.70257 PMC12158543

[tpj71031-bib-0025] Kui, L. , Chen, H. , Zhang, W. , He, S. , Xiong, Z. , Zhang, Y. et al. (2017) Building a genetic manipulation tool box for orchid biology: identification of constitutive promoters and application of CRISPR/Cas9 in the orchid, *Dendrobium officinale* . Frontiers in Plant Science, 7, 2036. 28127299 10.3389/fpls.2016.02036 PMC5226938

[tpj71031-bib-0026] Kuo, S.Y. , Hu, C.C. , Huang, Y.W. , Lee, C.W. , Luo, M.J. , Tu, C.W. et al. (2021) Argonaute 5 family proteins play crucial roles in the defence against cymbidium mosaic virus and *Odontoglossum* ringspot virus in *Phalaenopsis aphrodite* subsp. *formosana* . Molecular Plant Pathology, 22, 627–643. 33749125 10.1111/mpp.13049 PMC8126185

[tpj71031-bib-0027] Lee, S.C. , Pai, H. , Huang, Y.W. , He, M.H. , Song, Y.L. , Kuo, S.Y. et al. (2021) Exploring the multifunctional roles of *Odontoglossum* ringspot virus p126 in facilitating cymbidium mosaic virus cell‐to‐cell movement during mixed infection. Viruses, 13, 1552. 34452417 10.3390/v13081552 PMC8402721

[tpj71031-bib-0028] Liu, Q. , Zhao, C. , Sun, K. , Deng, Y. & Li, Z. (2023) Engineered biocontainable RNA virus vectors for non‐transgenic genome editing across crop species and genotypes. Molecular Plant, 16, 616–631. 36751129 10.1016/j.molp.2023.02.003

[tpj71031-bib-0029] Lu, H.C. , Chen, C.E. , Tsai, M.H. , Wang, H.I. , Su, H.J. & Yeh, H.H. (2009) Cymbidium mosaic potexvirus isolate‐dependent host movement systems reveal two movement control determinants and the coat protein is the dominant. Virology, 388, 147–159. 19345971 10.1016/j.virol.2009.02.049 PMC7103407

[tpj71031-bib-0030] Ma, X. , Li, X. & Li, Z. (2023) Transgene‐free genome editing in *Nicotiana benthamiana* with CRISPR/Cas9 delivered by a rhabdovirus vector. In: Plant genome engineering: methods and protocols. New York, NY: Springer US, pp. 173–185. 10.1007/978-1-0716-3131-7_11 36995626

[tpj71031-bib-0031] Ma, X. , Zhang, X. , Liu, H. & Li, Z. (2020) Highly efficient DNA‐free plant genome editing using virally delivered CRISPR–Cas9. Nature Plants, 6, 773–779. 32601419 10.1038/s41477-020-0704-5

[tpj71031-bib-0032] Mao, Y. , Botella, J.R. , Liu, Y. & Zhu, J.K. (2019) Gene editing in plants: progress and challenges. National Science Review, 6, 421–437. 34691892 10.1093/nsr/nwz005 PMC8291443

[tpj71031-bib-0033] Mikami, M. , Toki, S. & Endo, M. (2017) In planta processing of the SpCas9–gRNA complex. Plant and Cell Physiology, 58, 1857–1867. 29040704 10.1093/pcp/pcx154 PMC5921533

[tpj71031-bib-0034] Mikhaylova, E. (2025) Virus‐induced genome editing (VIGE): one step away from an agricultural revolution. International Journal of Molecular Sciences, 26, 4599. 40429744 10.3390/ijms26104599 PMC12111327

[tpj71031-bib-0035] Nagalakshmi, U. , Meier, N. , Liu, J.Y. , Voytas, D.F. & Dinesh‐Kumar, S.P. (2022) High‐efficiency multiplex biallelic heritable editing in Arabidopsis using an RNA virus. Plant Physiology, 189, 1241–1245. 35389493 10.1093/plphys/kiac159 PMC9237674

[tpj71031-bib-0036] Nagalakshmi, U. , Rodriguez, J.E. , Nguyen, T. , Weissman, R.F. , Thornton, B.W. , Terrace, C.I. et al. (2026) High‐efficiency, transgene‐free plant genome editing by viral delivery of an engineered TnpB. Nature Plants, 12, 503–511. Available from: 10.1038/s41477-026-02237-4 41720886 PMC13013028

[tpj71031-bib-0037] Naito, Y. , Hino, K. , Bono, H. & Ui‐Tei, K. (2015) CRISPRdirect: software for designing CRISPR/Cas guide RNA with reduced off‐target sites. Bioinformatics, 31, 1120–1123. 25414360 10.1093/bioinformatics/btu743 PMC4382898

[tpj71031-bib-0038] Oh, Y. , Kim, H. & Kim, S.G. (2021) Virus‐induced plant genome editing. Current Opinion in Plant Biology, 60, 101992. 33450609 10.1016/j.pbi.2020.101992

[tpj71031-bib-0039] Oh, Y. , Nagalakshmi, U. , Dahlbeck, D. , Koehler, N. , Cho, M.J. , Dinesh‐Kumar, S.P. et al. (2025) Heritable virus‐induced germline editing in tomato. The Plant Journal, 122, e70115. 40163287 10.1111/tpj.70115 PMC11956848

[tpj71031-bib-0040] Semiarti, E. , Nopitasari, S. , Setiawati, Y. , Lawrie, M.D. , Purwantoro, A. , Widada, J. et al. (2020) Application of CRISPR/Cas9 genome editing system for molecular breeding of orchids. Indonesian Journal of Biotechnology, 25, 61–68.

[tpj71031-bib-0041] Shen, Y. , Ye, T. , Li, Z. , Kimutai, T.H. , Song, H. , Dong, X. et al. (2024) Exploiting viral vectors to deliver genome editing reagents in plants. aBIOTECH, 5, 247–261. 38974861 10.1007/s42994-024-00147-7 PMC11224180

[tpj71031-bib-0042] Steinberger, A.R. & Voytas, D.F. (2025) Virus‐induced gene editing free from tissue culture. Nature Plants, 11, 1241–1251. 40562813 10.1038/s41477-025-02025-6

[tpj71031-bib-0043] Tiwari, P. , Sharma, A. , Bose, S.K. & Park, K.I. (2024) Advances in orchid biology: biotechnological achievements, translational success, and commercial outcomes. Horticulturae, 10, 152.

[tpj71031-bib-0044] Tong, C.G. , Wu, F.H. , Yuan, Y.H. , Chen, Y.R. & Lin, C.S. (2019) High‐efficiency CRISPR/Cas‐based editing of *Phalaenopsis* orchid MADS genes. Plant Biotechnology Journal, 18, 889–891. 31553827 10.1111/pbi.13264 PMC7061860

[tpj71031-bib-0045] Uranga, M. , Aragonés, V. , Selma, S. , Vázquez‐Vilar, M. , Orzáez, D. & Daròs, J.A. (2021) Efficient Cas9 multiplex editing using unspaced sgRNA arrays engineering in a potato virus X vector. The Plant Journal, 106, 555–565. 33484202 10.1111/tpj.15164 PMC8251967

[tpj71031-bib-0046] Uranga, M. & Daròs, J.A. (2023) Tools and targets: the dual role of plant viruses in CRISPR‐Cas genome editing. Plant Genome, 16, e20220. 35698891 10.1002/tpg2.20220 PMC12806914

[tpj71031-bib-0047] Varanda, C.M. , Félix, M.D.R. , Campos, M.D. , Patanita, M. & Materatski, P. (2021) Plant viruses: from targets to tools for CRISPR. Viruses, 13, 141. 33478128 10.3390/v13010141 PMC7835971

[tpj71031-bib-0048] Verchot‐Lubicz, J. (2005) A new cell‐to‐cell transport model for potexviruses. Molecular Plant‐Microbe Interactions, 18, 283–290. 15828680 10.1094/MPMI-18-0283

[tpj71031-bib-0049] Voytas, D.F. & Gao, C. (2014) Precision genome engineering and agriculture: opportunities and regulatory challenges. PLoS Biology, 12, e1001877. 24915127 10.1371/journal.pbio.1001877 PMC4051594

[tpj71031-bib-0050] Wang, Y. , Zafar, N. , Ali, Q. , Manghwar, H. , Wang, G. , Yu, L. et al. (2022) CRISPR/Cas genome editing technologies for plant improvement against biotic and abiotic stresses: advances, limitations, and future perspectives. Cells, 11, 3928. 36497186 10.3390/cells11233928 PMC9736268

[tpj71031-bib-0051] Weiss, T. , Kamalu, M. , Shi, H. , Li, Z. , Amerasekera, J. , Zhong, Z. et al. (2025) Viral delivery of an RNA‐guided genome editor for transgene‐free germline editing in Arabidopsis. Nature Plants, 11, 967–976. 40263581 10.1038/s41477-025-01989-9 PMC12095077

[tpj71031-bib-0052] Wong, S.M. , Mahtani, P.H. , Lee, K.C. , Yu, H.H. , Tan, Y. , Neo, K.K. et al. (1997) Cymbidium mosaic potexvirus RNA: complete nucleotide sequence and phylogenetic analysis. Archives of Virology, 142, 383–391. 9125051 10.1007/s007050050084

[tpj71031-bib-0053] Wu, L. , Gu, Y. , Guo, H. , Zhang, J. , Yang, J. , Zhang, M. et al. (2025) BaMV‐vectored compact AsCas12f1‐HKRA enables transgene‐free genome editing in Moso bamboo (*Phyllostachys edulis*). Plant Biotechnology Journal, 24, 1–3. 10.1111/pbi.70474 PMC13140604 41329479

[tpj71031-bib-0054] Wu, X. , Zhang, Y. , Jiang, X. , Ma, T. , Guo, Y. , Wu, X. et al. (2024) Considerations in engineering viral vectors for genome editing in plants. Virology, 589, 109922. 37924727 10.1016/j.virol.2023.109922

[tpj71031-bib-0055] Xing, H.L. , Dong, L. , Wang, Z.P. , Zhang, H.Y. , Han, C.Y. , Liu, B. et al. (2014) A CRISPR/Cas9 toolkit for multiplex genome editing in plants. BMC Plant Biology, 14, 327. 25432517 10.1186/s12870-014-0327-y PMC4262988

[tpj71031-bib-0056] Xu, F. & Copeland, C. (2012) Nuclear extraction from *Arabidopsis thaliana* . Bio‐Protocol, 2, e306.

[tpj71031-bib-0057] Yin, K. , Han, T. , Liu, G. , Chen, T. , Wang, Y. , Yu, A.Y.L. et al. (2015) A geminivirus‐based guide RNA delivery system for CRISPR/Cas9 mediated plant genome editing. Scientific Reports, 5, 14926. 26450012 10.1038/srep14926 PMC4598821

[tpj71031-bib-0058] Yusop, M.S.M. , Mohamed‐Hussein, Z.A. , Ramzi, A.B. & Bunawan, H. (2022) Cymbidium mosaic virus infecting orchids: what, how, and what next? Iranian Journal of Biotechnology, 20, e3020. 35891960 10.30498/ijb.2021.278382.3020 PMC9284244

[tpj71031-bib-0059] Zhang, C. , Liu, S. , Li, X. , Zhang, R. & Li, J. (2022) Virus‐induced gene editing and its applications in plants. International Journal of Molecular Sciences, 23, 10202. 36142116 10.3390/ijms231810202 PMC9499690

[tpj71031-bib-0060] Zhu, H. , Li, C. & Gao, C. (2020) Applications of CRISPR–Cas in agriculture and plant biotechnology. Nature Reviews Molecular Cell Biology, 21, 661–677. 32973356 10.1038/s41580-020-00288-9

