# Supplementary material for: Efficient CRISPR‐Cas9 delivery and transgene‐free multiplex genome editing in plants using cymbidium mosaic virus‐derived vectors
Source: Plant J. 2026 Jul 6;127(1):e71031. doi: 10.1111/tpj.71031 (PMC13337336; doi:10.1111/tpj.71031)
Supplement: Supplementary file 1 — Figure S1. Schematic representation of the NbPDS gene structure in the allotetraploid N. benthamiana, comprising two homoeologs, NbPDS‐A and NbPDS‐B. Figure S2. RT‐PCR analysis of gRNA‐associated RNA species in total and nuclear‐enriched RNA fractions. Figure S3. The in cis Cas9‐gRNA expression strategy enhances transient genome editing efficiency. Figure S4. ORSV P126 enhances CymMV accumulation and systemic expression in orchids. Figure S5. Target site design for multiplex genome editing in P. aphrodite. Figure S6. Conservation of the PaPDS gRNA target site among Phalaenopsis species and validation of CymMV‐mediated genome editing in Phalaenopsis equestris. Table S1. List of primers used in this study. [file TPJ-127-0-s001.pdf]

Supplemental Table S1 List of primers used in this study.

**Primers for construction**		
Construct name	Primer name	Primer sequence (5′→3′)
pKCyCas9-NbPDSgRNA	PacI/NbPDSgRNA_F	GCTTAATTAATTGGTAGTAGCGACTCCATGGTTTTAGAGCTAG
	PacI/gRNA_R	GCTTAATTAAGCACCGACTCGGTGCC
pKCyCas9-PaPDSgRNA	PacI-PmlI/PaPDSgRNA1-F	GCTTAATTAATTGACACGTGCCAGCAATTACCACTTCTAG
	PacI/gRNA_R	GCTTAATTAAGCACCGACTCGGTGCC
pKCyCas9-PaPDS2+1gRNA	PacI/PaPDSgRNA2_F	GCTTAATTAAGCAGTGGAAGGAACATTCCAGTTTTAGAGCTAG
	PacI/gRNA_R	GCTTAATTAAGCACCGACTCGGTGCC
pKCydCCas9	PacI/Cy3'UTR_F	GCTTAATTAACGCCAAACTTAATAAG
	SacI/oligodT21	GCGAGCTCTTTTTTTTTTTTTTTTTTTTT
**Primers for RT-PCR**		
Gene name	Primer name	Primer sequence (5′→3′)
NbPDSgRNA	PDSgRNA_F	TTGGTAGTAGCGACTCCATG
	PDSgRNA_R	GCACCGACTCGGTGCCAC
U6 SnRNA	U6snRNA_F	GTCCCTTCGGGGACATCCGATA
	U6snRNA_R	TTGGACCATTTCTCGATTTGTG
Cas9-CP	Cas9-4130F	GCACTAAGGAGGTCCTGG
	Cyy1-6217R	GGCTAAACATATTATGCCAGTAGTG
**Primers used in mutation detection**		
Target loci	Primer name	Primer sequence (5′→3′)
NbPDS	NbPDS_geF	GTCAGGCTTAATTTACTGC
	NbPDS_geR	CGCATTCAAAACAAACCTTTAAAG
PaPDS1	PaPDS1_geF	GTGCAGGTTTTCTGCATG
	PaPDS1_geR	CAGAACATCTCTTGCCTC
PaPDS2	PaPDS2_geF	CATCCCTACAGTTTAATTAGTAG
	PaPDS2_geR	CCTCAAACAAACCAACTC

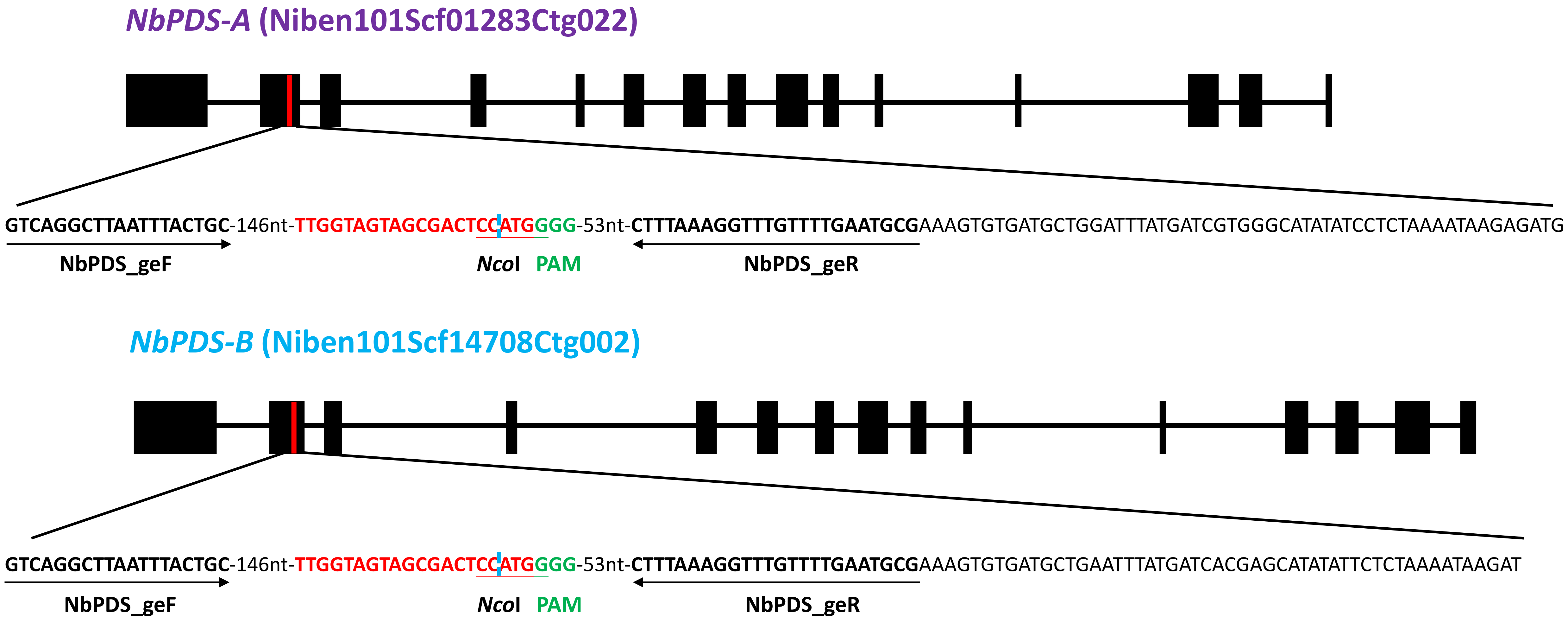

FIGURE S1 Schematic representation of the *NbPDS* gene structure in the allotetraploid *N. benthamiana*, comprising two homoeologs, *NbPDS-A* and *NbPDS-B*. The selected gRNA target sequence (red) and the Protospacer Adjacent Motif (PAM, green) are conserved across both homoeologs. An *Nco*I restriction site (underlined) overlapping the Cas9 cleavage site was identified for downstream mutation detection assays.

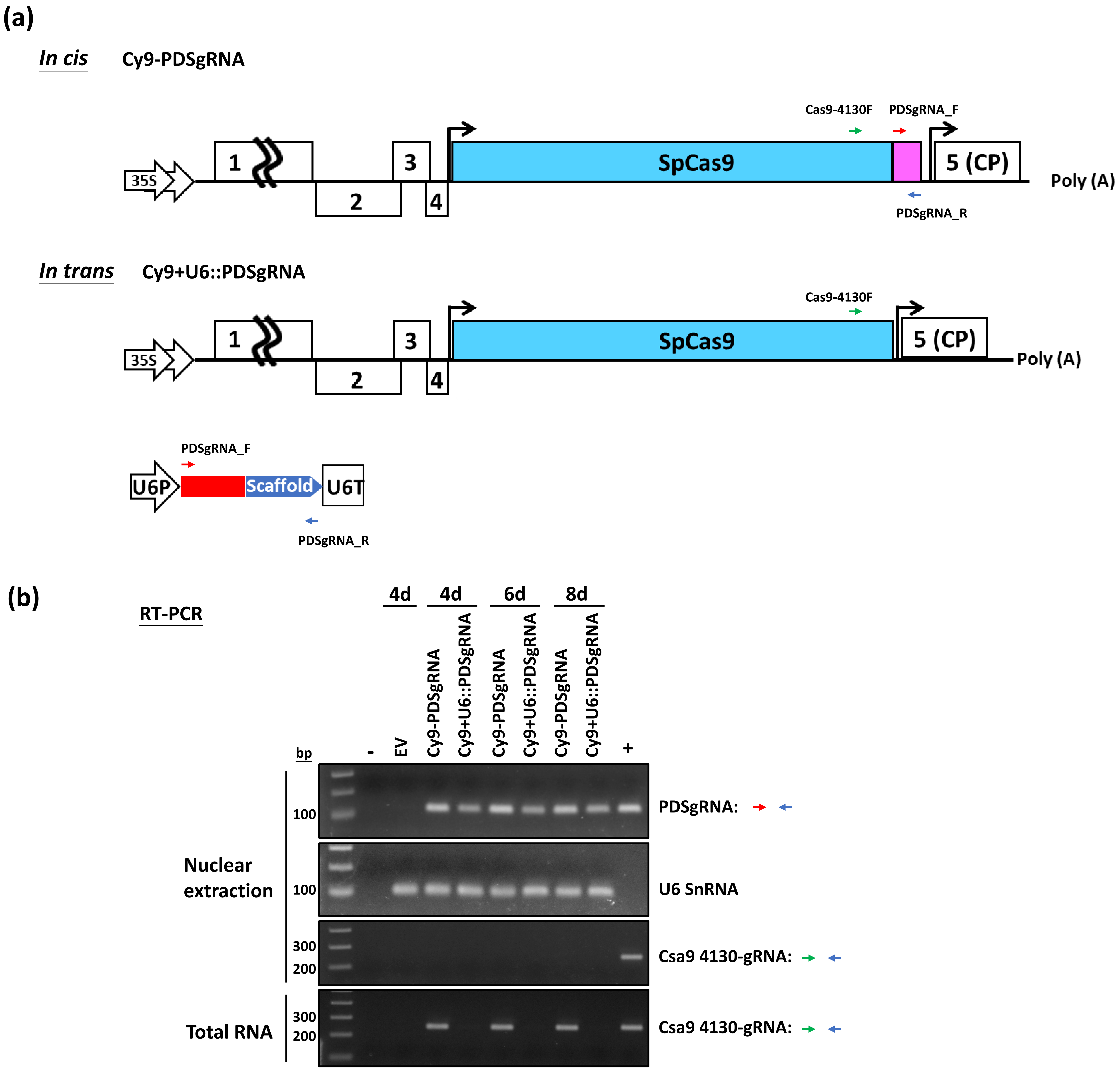

FIGURE S2 RT-PCR analysis of gRNA-associated RNA species in total and nuclear-enriched RNA fractions. (a) Schematic representation of viral vectors used for the *in cis* and *in trans* delivery strategies, with primer positions used for RT-PCR analysis indicated. Reverse transcription was performed using the PDSsgRNA_R primer. Colored arrows indicate primer pairs targeting the PDSgRNA region or upstream Cas9-linked viral RNA regions. (b) RT-PCR analysis of total RNA and nuclear-enriched RNA fractions extracted from infiltrated *N. benthamiana* leaves. The infection treatment and sampling time point are indicated above each lane. Water was used as the negative control (-), and the infectious clone pKCyCas9-NbPDSgRNA was used as the positive control (+) for PCR.

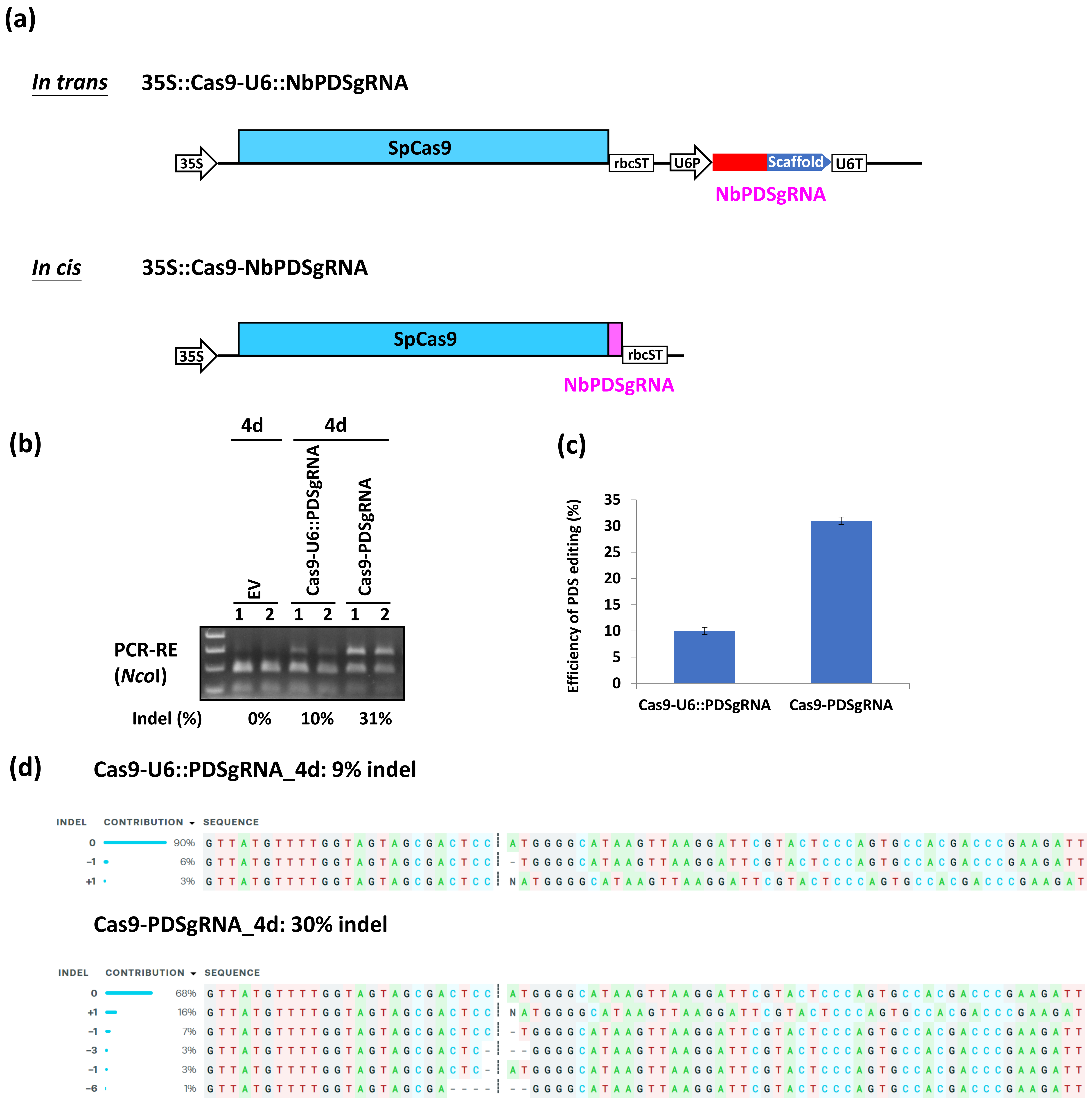

FIGURE S3 The *in cis* Cas9-gRNA expression strategy enhances transient genome editing efficiency. (a) Schematic representation of transient expression constructs expressing Cas9 and NbPDS gRNA in either an *in trans* (35S::Cas9-U6::NbPDSgRNA) or *in cis* (35S::Cas9-NbPDSgRNA) way. (b) PCR-RE analysis of *NbPDS* target sites at 4 dpi. Indel percentages calculated by ICE analysis are shown below the gels. (c) Quantification of *NbPDS* editing efficiency from three independent biological replicates. (d) Indel profiles determined by Sanger sequencing and ICE analysis, showing increased mutation frequency and diversity in the *in cis* expression strategy.

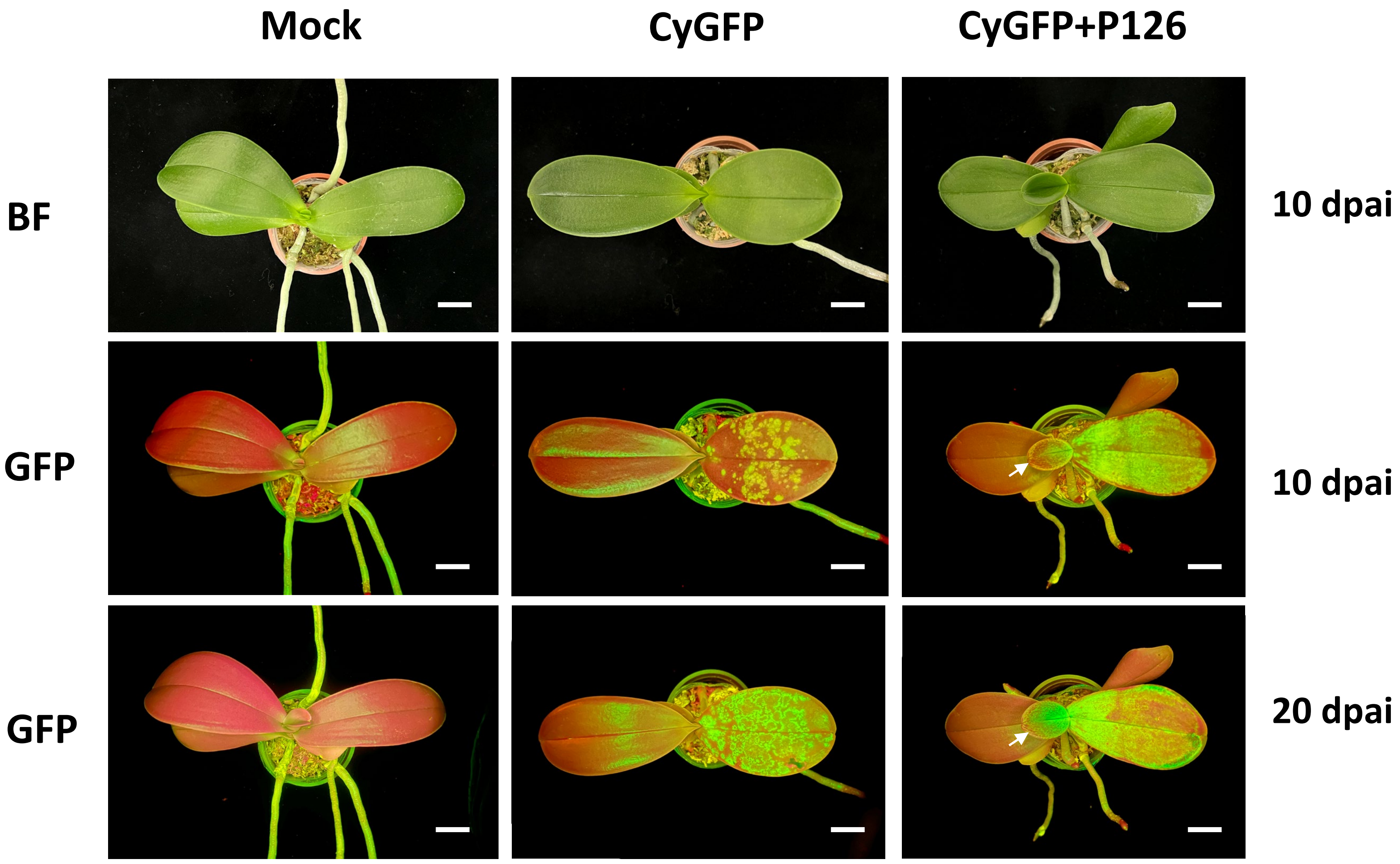

FIGURE S4 ORSV P126 enhances CymMV accumulation and systemic expression in orchids. Bright-field (BF) and GFP fluorescence images of orchid plants infected with CymMV-GFP alone or co-expressed with the ORSV P126 silencing suppressor at 10 and 20 days post agroinfiltration. White arrows indicate GFP fluorescence detected in non-inoculated upper leaves. Scale bar = 2 cm.

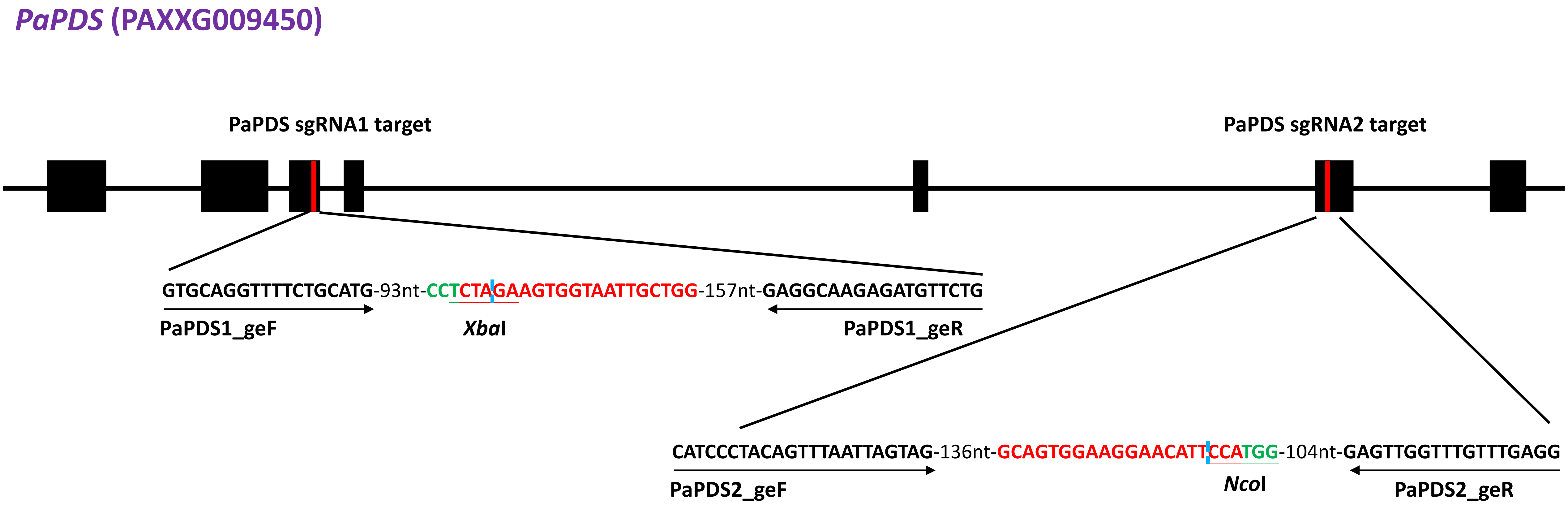

FIGURE S5 Target site design for multiplex genome editing in *P. aphrodite*. Partial genomic sequence of the *PaPDS* gene showing the locations and sequences of the two selected target sites, PaPDS1 (containing an *Xba*I site) and PaPDS2 (containing an *Nco*I site), along with the primers used for amplification.

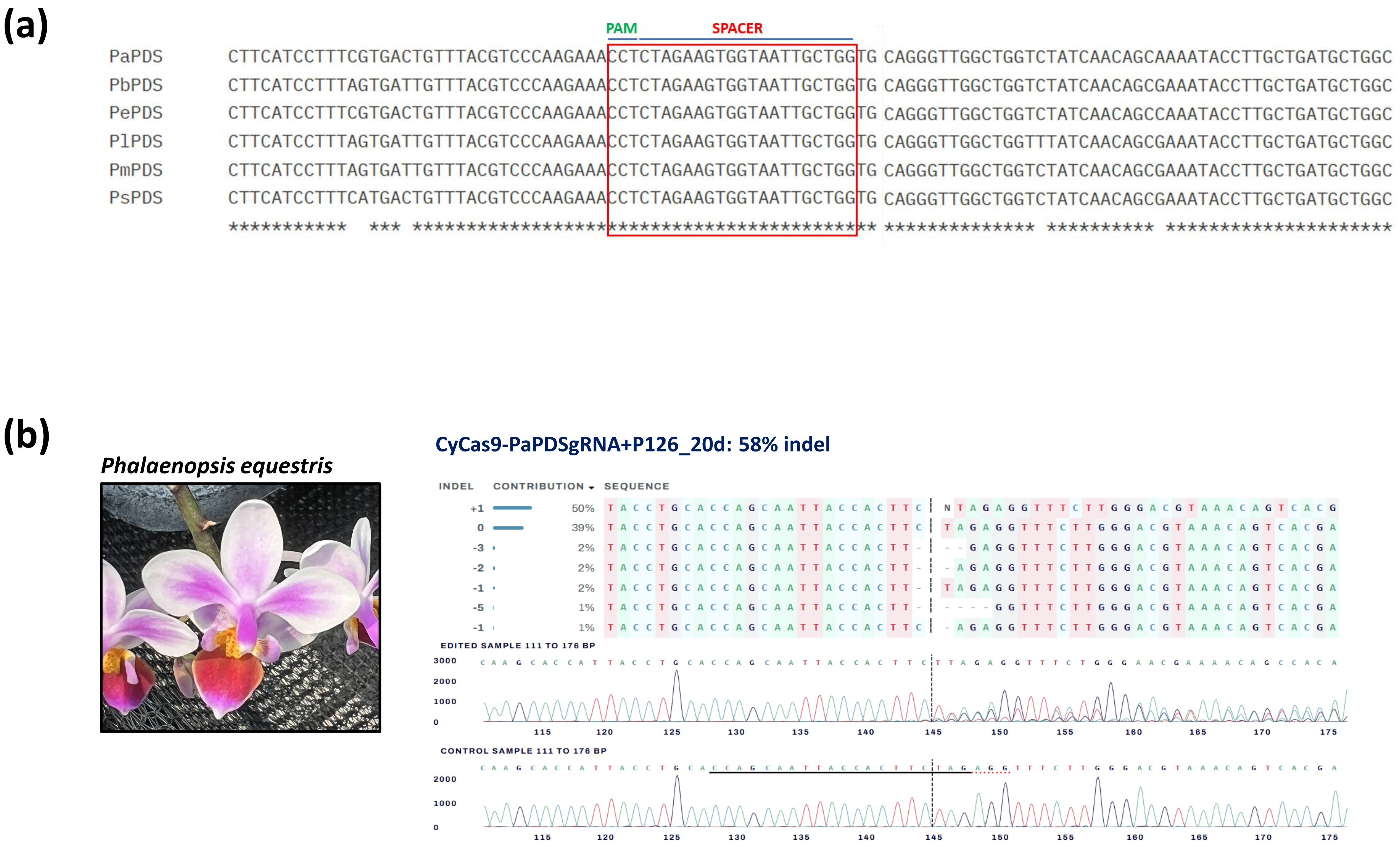

FIGURE S6 Conservation of the PaPDS gRNA target site among *Phalaenopsis* species and validation of CymMV-mediated genome editing in *Phalaenopsis equestris*. (a) Multiple sequence alignment of *PaPDS* target regions from different *Phalaenopsis* species, showing that the gRNA target sequence is identical among species. Pa, *P. aphrodite*; Pb, *P. bellina*; Pe, *P. equestris*; Pl, *P. lueddemanniana*; Pm, *P. modesta*; Ps, *P. schilleriana*. (b) ICE analysis of genome editing efficiency in *Phalaenopsis equestris* leaves co-infiltrated with CyCas9-PaPDSgRNA and the P126 silencing suppressor at 20 dpi.
